# Cross-priming induces immunodomination in the presence of viral MHC class I inhibition

**DOI:** 10.1371/journal.ppat.1006883

**Published:** 2018-02-14

**Authors:** Elvin J. Lauron, Liping Yang, Jabari I. Elliott, Maria D. Gainey, Daved H. Fremont, Wayne M. Yokoyama

**Affiliations:** 1 Department of Medicine, Washington University School of Medicine, St. Louis, Missouri, United States of America; 2 Department of Pathology and Immunology, Washington University School of Medicine, St. Louis, Missouri, United States of America; 3 Department of Biology, Western Carolina University, Cullowhee, North Carolina, United States of America; 4 Department of Biochemistry and Molecular Biophysics, Washington University School of Medicine, St. Louis, Missouri, United States of America; 5 Department of Molecular Microbiology, Washington University School of Medicine, St. Louis, Missouri, United States of America; 6 Division of Rheumatology, Washington University School of Medicine, St. Louis, Missouri, United States of America; McMaster University, CANADA

## Abstract

Viruses have evolved mechanisms of MHCI inhibition in order to evade recognition by cytotoxic CD8^+^ T cells (CTLs), which is well-illustrated by our prior studies on cowpox virus (CPXV) that encodes potent MHCI inhibitors. Deletion of CPXV viral MHCI inhibitors markedly attenuated *in vivo* infection due to effects on CTL effector function, not priming. However, the CTL response to CPXV in C57BL/6 mice is dominated by a single peptide antigen presented by H-2K^b^. Here we evaluated the effect of viral MHCI inhibition on immunodominant (IDE) and subdominant epitopes (SDE) as this has not been thoroughly examined. We found that cross-priming, but not cross-dressing, is the main mechanism driving IDE and SDE CTL responses following CPXV infection. Secretion of the immunodominant antigen was not required for immunodominance. Instead, immunodominance was caused by CTL interference, known as immunodomination. Both immunodomination and cross-priming of SDEs were not affected by MHCI inhibition. SDE-specific CTLs were also capable of exerting immunodomination during primary and secondary responses, which was in part dependent on antigen abundance. Furthermore, CTL responses directed solely against SDEs protected against lethal CPXV infection, but only in the absence of the CPXV MHCI inhibitors. Thus, both SDE and IDE responses can contribute to protective immunity against poxviruses, implying that these principles apply to poxvirus-based vaccines.

## Author summary

The use of vaccinia virus (VACV) to eradicate smallpox is the arguably the most successful demonstration of vaccination. The VACV vaccine also provides cross-protection against related zoonotic orthopoxviruses, including monkey poxvirus (MXPV) and CPXV, which circulate between various animal hosts and humans. Interestingly, Edward Jenner first demonstrated the concept of vaccination against smallpox in the late 1700s using CPXV. He also made the curious observation that CPXV vaccination did not always protect against recurrent exposure to CPXV. Jenner’s observations may be explained by the ability for CPXV to evade antiviral CD8^+^ T cell immune responses. To evade CD8^+^ T cells, CPXV inhibits MHCI antigen presentation, which is required to prime CD8^+^ T cells. Importantly, CPXV is the only orthopoxvirus that inhibits MHCI and thus provides a unique opportunity to investigate the effects of viral MHCI inhibition on CD8^+^ T cell priming. Here, we examine the factors that contribute to priming of CPXV-specific CD8^+^ T cells and show that viral MHCI inhibition does not affect CD8^+^ T cell priming, but prior CPXV immunization does inhibit priming during subsequent exposure to CPXV. The effects of pre-existing poxvirus immunity are therefore important to consider if poxvirus-based vaccines against various diseases are to be widely used.

## Introduction

Strategies to leverage strong cytotoxic CD8^+^ T cells (CTL) responses to viral infections are of particular interest as CTLs play essential roles in controlling viral infections [1–5]. Before gaining effector functions, virus-specific CTL precursors must be primed by antigen presenting cells (APCs) that present pathogen-derived epitopes via major histocompatibility complex class I (MHCI) molecules on the cell surface. If the APC is infected and directly presents endogenously produced antigens, this is known as direct presentation. Alternatively, uninfected APCs may process and cross-present exogenous antigens from infected cells. Cross-presentation is mediated primarily by Batf3-dependent CD103^+^/CD8α^+^ dendritic cells (DCs) [6–8], which we refer to as BATF3^+^ DCs. Peptide-loaded MHCI molecules from infected cells may also be liberated by cell lysis or secreted in exosomes and then transferred onto cross-presenting APCs. When uninfected APCs acquire preformed peptide-MHCI complexes in this manner, they are termed cross-dressed and can drive expansion of CD8^+^ T cells [9–11]. Induction of CD8^+^ T cell responses by cross-dressing was previously demonstrated in studies using adoptive transfer of T cell receptor (TCR) transgenic (Tg) T cells [9–11] and also requires BATF3^+^ DCs [11]. However, the relative contribution of these processes to non-TCR Tg CTL responses against viral antigens is largely unknown.

Upon recognizing cognate antigen on APCs, naïve CTLs are activated to undergo clonal expansion and traffic to the site of ongoing viral infection. There, virus-specific CTLs mediate host resistance by recognizing infected cells via surface MHCI molecules displaying processed viral antigens. Specific T cell recognition activates direct killing of infected cells and production of interferon-gamma (IFN-γ) and other cytokines that may have indirect effects. In the later stages of the response, a proportion of CTLs become long-lived memory CD8^+^ T cells that can provide rapid protection during secondary responses to the viral pathogens.

Many viruses display mechanisms that may contribute to evading CTL responses, such as inhibiting MHCI antigen presentation. The effects and mechanisms of MHCI inhibition on CTL responses have been well demonstrated *in vitro* with herpesviruses [12]. For instance, downregulation of MHCI by murine cytomegalovirus (MCMV) prevented MCMV-specific CTLs from killing infected cells, whereas cells infected with an MCMV mutant lacking the viral MHCI inhibitors were lysed by CTLs [13]. However, the *in vivo* relevance of viral MHCI inhibition in general was previously unclear since herpesvirus-mediated MHCI inhibition had few effects on *in vivo* CTL responses in murine and nonhuman primate infection models [14–16].

On the other hand, studies of cowpox virus (CPXV) by our lab and others indicated that CPXV, uniquely among the orthopoxviruses, mediated mouse and human MHCI inhibition by two open reading frames (ORFs), CPXV012 and CPXV203 [4,17,18]. CPXV203 retains MHCI molecules in the ER and CPXV012 inhibits peptide loading on MHCI molecules; when combined, these two evasion mechanisms allows CPXV to evade CTL responses. The deletion of intact CPXV012 and CPXV203 from the CPXV genome attenuated viral pathogenesis *in vivo* [4,5]. Furthermore, this attenuation was dependent on the anti-CPXV CTL response since depleting CD8^+^ T cells restored the virulence of the Δ12Δ203 CPXV mutant, similar to wild type (WT) CPXV. Thus, these studies of CPXV established the *in vivo* importance of viral MHCI inhibition and its effects on antiviral CTL responses.

Interestingly, the virus-specific CTL response to CPXV in C57BL/6 mice is dominated by a single antigen (B8), displaying the immunological phenomenon known as immunodominance, that can impede the development of efficacious vaccines [19]. In theory, removing the IDE(s) may circumvent immunity. However, for some viruses, subdominant epitopes (SDEs) may compensate and then dominate the immune response [20,21]. Such findings revealed that responses against an IDE(s) suppress immune responses to SDEs, which is a related yet distinct phenomenon coined immunodomination. CD8^+^ T cell immunodomination also occurs during secondary responses whereby memory CD8^+^ T cells can suppress naïve CD8^+^ T cell responses [22]. CD8^+^ T cell immunodomination is likely a mechanism that contributes to the immunodominance of the B8 antigen in CPXV infections [23], but has not been studied in the context of MHCI inhibition.

*B8R* is a highly conserved gene among orthopoxviruses and encodes the secreted soluble B8 protein that binds IFN-γ with broad species-specificity. B8 from ectromelia virus (ECTV) is a strong inhibitor of human, bovine, rat, and murine IFN-γ [24], but VACV and CPXV B8 does not neutralize murine IFN-γ [25]. These differences have been attributed to host-specificity. While the natural host of ECTV is not known, experimentally it is restricted to murine hosts, whereas VACV has a broad host-tropism with an unknown natural reservoir [26]. The natural reservoirs of CPXV are wild-rodent species, but CPXV also has broad host-tropism [27,28]. Despite these differences, B8 is the most dominant antigen identified in mice with the H-2K^b^ MHCI allele, and the B8 CD8^+^ T cell epitope sequence (TSYKFESV) is 100% conserved between ECTV, VACV, CPXV, and other orthopoxviruses [29]. However, it is not clear if B8 is an immunodominant antigen because it is a secreted soluble protein that may be efficiently cross-presented.

Previously, we showed that CPXV infection of *Batf3*^*-/-*^ mice that selectively lack the main cross-presenting DC subsets (CD103^+^/CD8α^+^ DCs) [30] display reduced priming of B8_19-26_-specific CD8^+^ T cells during CPXV infection [5], suggesting that cross-presentation is a major pathway used to induce CTLs. However, since *Batf3*^*–/–*^mice also lack the capability of cross-dressing, it is also possible that cross-dressing is the main pathway to induce CPXV-specific CTLs. Moreover, it remained unclear whether other CPXV antigens (i.e., SDEs) are efficiently presented by BATF3^+^ DCs because CPXV B8_19-26_ immunodominates the primary CTL response [5]. Finally, due to the above limitations, it is not known if these processes could be affected by viral MHCI inhibition.

Here we studied if transmembrane anchoring of B8 affects its immunodominance, the role of MHCI inhibition in the generation of virus-specific CTLs to SDEs and for the first time, the relevance of cross-dressing in the induction of endogenous antiviral CTL responses.

## Results

### Secretion of the immunodominant antigen is not a determinant for immunodominance

**Fig 1:**
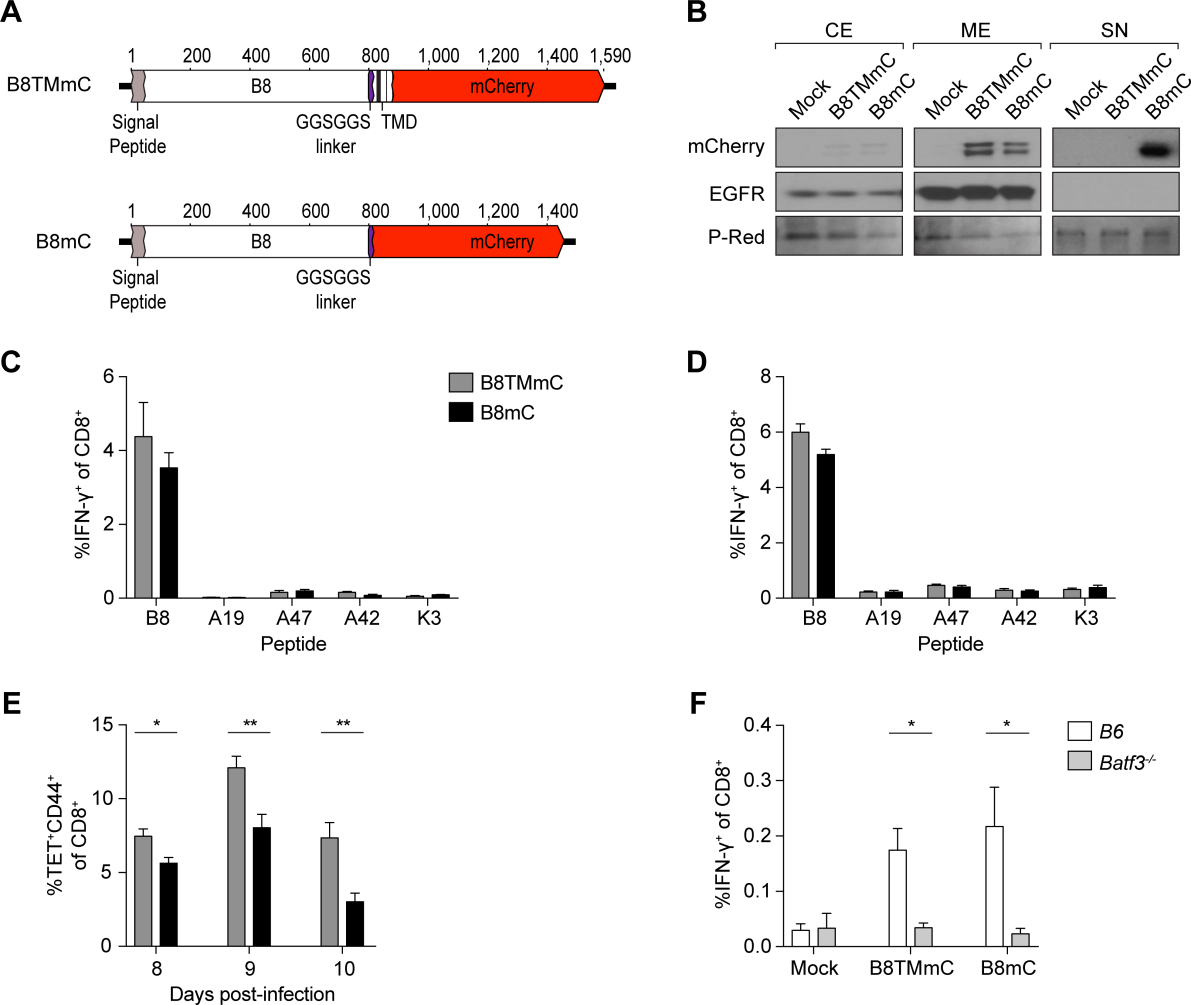
Secretion of the immunodominant antigen is not required for immunodominance. (A) Schematic representation of the B8-mCherry fusion proteins; the location of the signal peptide, GGSGGS linker, TMD, and mCherry are depicted. (B) B8TMmC is not secreted. HeLa cells were infected at an MOI of 5 with B8TMmC or B8mC. Cells and supernatant were harvested at 4 hpi for subcellular fractionation and mCherry and EGFR expression was determined by western blot; equal loading and transfer of samples was confirmed with ponceau S red (P-Red) staining. CE = cytoplasmic extract; ME = membrane extract; SN = supernatant. Data are representative of two independent experiments. (C, D) Comparable CTL priming by B8TMmC and B8mC. CD8^+^ T cell responses in the spleen of B6 (n = 5) i.n. infected with 5 x 10^3^ pfu (C) and 1.5 x 10^4^ pfu (D) B8TMmC or B8mC were determined by *ex vivo* restimulation with CPXV peptides and ICS at 8 dpi. Data are representative of two independent experiments. (E) Cell-associated antigen is cross-presented more efficiently than soluble antigen. B8-specific CD8^+^ T cell responses in the spleen of B6 (n = 5) i.n. infected with 1.5 x 10^5^ pfu B8TMmC or B8mC were determined by tetramer staining at 8, 9, and 10 dpi. Data are representative of two independent experiments. (F) CD8^+^ T cell responses require BATF3^+^ DCs. B6 and *Batf3*^*-/-*^ mice (n = 7–10) were i.n. infected with 5 x 10^3^ pfu B8TMmC or B8mC and the B8-specific CD8^+^ T cell responses in the spleen were determined at 6 dpi. n = 3 mock-infected mice. Data are the combined results of three independent experiments.

The immunodominant CPXV B8 antigen is a secreted soluble protein [24], suggesting that its immunodominance may be due to its property as a secreted molecule, as shown for other antigens [31,32]. If this were true, we expect that altering the protein targeting of B8 so that it is no longer secreted from infected cells will affect the acquisition and availability of B8 for APCs, which in turn would affect priming of B8_19-26_-specific CD8^+^ T cells and its immunodominance. To test these hypotheses and detect subcellular location of B8, we produced a CPXV mutant expressing B8 fused to mCherry (B8mC) and another mutant (B8TMmC) expressing B8-mCherry fusion protein with a transmembrane domain (TMD) (Fig 1A).

We performed subcellular fractionation of infected HeLa cells and analyzed the cytoplasmic extract, membrane extract, and supernatant by Western blot to determine the subcellular location of the B8 variants and if they were secreted. The B8 variants were mainly detected in the membrane extract of both B8TMmC- and B8mC-infected cells, indicating that the infected cells successfully expressed both B8 variants (Fig 1B). We note that the membrane fraction may contain proteins found within the mitochondria and endoplasmic reticulum, but not nuclear proteins, such that the secreted B8 variant detected in the membrane fraction is likely due to proteins localized within the ER and in transit through the secretory pathway. We also detected higher levels of the non-secreted B8 variant in the membrane fraction in comparison to the secreted variant, which is likely due to an accumulation of membrane-associated B8 within B8TMmC-infected cells. Most importantly, the B8 variant was detected in the supernatant of cells infected with B8mC, but not in the supernatant of cells infected with B8TMmC, demonstrating that the B8 variant remains cell-associated in cells infected with B8TMmC (Fig 1B). However, anchoring the B8 antigen did not negatively affect priming of B8_19-26_-specific CD8^+^ T cells and B8_19-26_ maintained the highest position in the immunodominance hierarchy, as shown in mice infected intranasally (i.n.) with B8TMmC or B8mC (Fig 1C and 1D). These data show that secretion of the B8 antigen is not required for priming of B8_19-26_-specific CD8^+^ T cells or immunodominance during CPXV infection.

We also performed kinetic analyses of B8_19-26_-specific CD8^+^ T cells by staining with H-2K^b^ tetramers loaded with B8_19-26_ peptide and found that priming by cell-associated B8 resulted in greater expansion of B8_19-26_-specific CD8^+^ T cells (Fig 1E). These results are consistent with previous findings that cell-associated antigens are cross-presented better than soluble antigens [33,34]. When we infected *Batf3*^*-/-*^ mice with B8TMmC or B8TM, we found that priming of B8_19-26_ -specific CD8^+^ T cells was significantly reduced in *Batf3*^*-/-*^ mice in comparison to B6 mice (Fig 1F), indicating that the introduced B8 mutations did not alter the dependence on cross-presentation (or cross-dressing) in the induction of B8_19-26_-specific CTL precursors. Since priming against the non-secreted B8 protein is still dependent on cross-presenting (or cross-dressed) BATF3^+^ DCs, it is likely that antigens used for conventional cross-presentation by BATF3^+^ DCs are acquired from infected apoptotic/necrotic donor cells or that BATF3^+^ DCs are cross-dressed with peptide-loaded MHCI molecules.

### Cross-presentation, but not cross-dressing of APCs, drives CTL responses during CPXV infection

**Fig 2:**
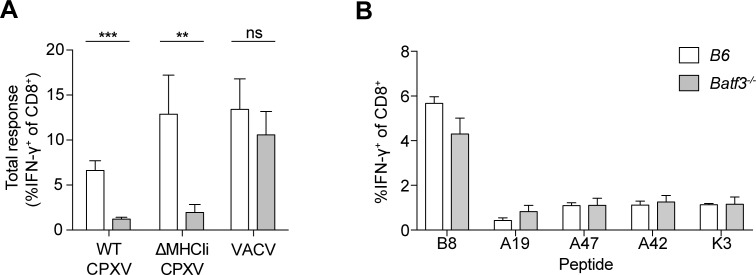
Cross-priming induces CTL responses during CPXV infection. (A) BATF3^+^ DCs cross-prime CPXV-specific CTL precursors. B6 or *Batf3*^*-/-*^ mice (n = 6) were infected i.p. with 1 x 10^5^ pfu WT CPXV, ΔMHCIi, or VACV-WR, and CD8^+^ T cell responses were measured by *ex vivo* restimulation with infected DC2.4 cells and ICS at 8 dpi. The data are the combined results of three independent experiments. (B) Induction of VACV-specific CTLs is not dependent BATF3^+^ DCs. B6 or *Batf3*^*-/-*^ mice (n = 6–7) were infected i.p. with 1 x 10^5^ pfu VACV-WR and CD8^+^ T cell responses in the spleen were measured at 6 dpi. The data are the combined results of two independent experiments.

While we previously reported that priming of CD8^+^ T cell responses to CPXV is dependent on cross-presenting BATF3^+^ DCs, others reported that direct priming is the main mechanism to induce CTL responses with VACV infection [35,36]. To directly compare these findings, we assessed the CTL response after systemic infection with WT CPXV, Δ12Δ203 (from here on referred to as ΔMHCIi) CPXV, or VACV in B6 and *Batf3*-deficient mice. At 8 days post-infection (dpi), the frequency of splenic CD8^+^ T cells that produced IFN-γ in *ex vivo* stimulations with ΔMHCIi-infected DC2.4 cells was significantly reduced in WT CPXV- and ΔMHCIi-infected *Batf3*^*-/-*^ mice (Fig 2A) in comparison to infected B6 mice, confirming the importance of cross-presentation (or cross-dressing) in inducing CPXV-specific CTLs, as we showed earlier [5]. Conversely, at 6 or 8 dpi, *ex vivo* stimulation with a set of 5 VACV/CPXV peptides (Fig 2B) or VACV-infected DC2.4 (Fig 2A) revealed no significant difference in the VACV-specific response between infected B6 and *Batf3*^*-/-*^ mice. These results are consistent with the findings that ablation of XCR1-expressing (CD103^+^/CD8α^+^) DCs does not completely abolish priming of CD8^+^ T cells during VACV infection [37]. Thus, the *in vivo* responses to two highly related orthopoxviruses display distinct requirements for direct presentation (VACV) versus cross-presentation/cross-dressing (CPXV).

**Fig 3:**
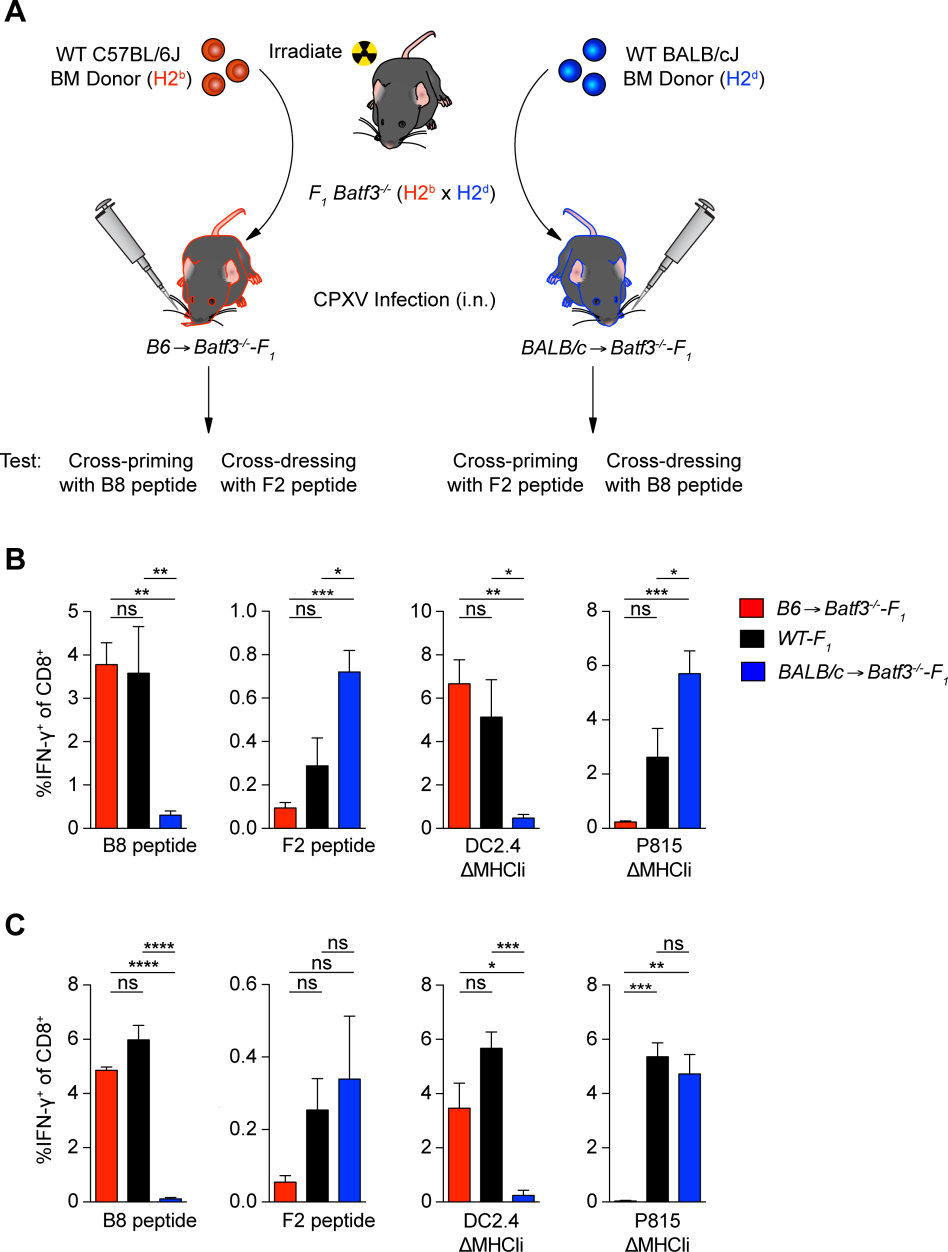
Conventional cross-priming, but not cross-dressing, is the main mechanism driving CTL responses during CPXV infection. (A) Schematic of bone marrow chimera cross-dressing experiment. B) CTLs are not activated by cross-dressed APCs. *Batf3*^*-/-*^-F_1_ mice (n = 5–6) were depleted of NK cells, lethally irradiated 2 days after NK cell depletion, and reconstituted with 1 x 10^7^ T cell depleted bone marrow cells from B6 or BALB/c mice. 8 weeks later, chimeric mice were infected i.n. with 5 x 10^3^ pfu WT CPXV and CD8^+^ T cell responses in the lungs were determined by ICS at 8 dpi. (C) Memory CTLs are not activated by cross-dressed APCs. Chimeric mice (n = 3–5) previously infected for 25 days were boosted with 5 x 10^4^ pfu WT CPXV and CD8^+^ T cell responses in the lungs were determined by ICS at 8 days after boost. The data are the combined results of three independent experiments.

Given that priming of CPXV-specific CTL precursors and cross-dressing of APCs in other settings were both shown to require BATF3^+^ DCs [11], we sought to determine if cross-dressing could account for the source of antigen being presented to CD8^+^ T cells in CPXV infection. To do so, we transferred B6 bone marrow (BM) into lethally irradiated *Batf3*^*-/-*^-F_1_ (*Batf3*^*-/-*^-B6 x *Batf3*^*-/-*^-BALB/c) mice (Fig 3A). In B6→*Batf3*^*-/-*^-F_1_ chimeras, donor B6-derived (Batf3-dependent) APCs only express H-2^b^ MHCI molecules and should cross-prime CTL responses against H2^b^-restricted epitopes (Fig 3A). However, priming by H-2^d^-restricted epitopes would occur only if the H2^b^ APCs in these chimeric mice were cross-dressed with preformed peptide-loaded H-2^d^ class I molecules from the host parenchymal cells, which express both H-2^b^ and H-2^d^ class I molecules. We also produced BALB/c→*Batf3*^*-/-*^-F_1_ chimeras, to analyze the converse situation. The reconstituted mice were infected by i.n. administration with WT CPXV and CTL responses were determined against the immunodominant H-2K^b^-restricted B8_19-26_ and the H-2L^d^-restricted F2_26-34_ epitopes. As expected, we detected a B8_19-26_ response in B6→*Batf3*^*-/-*^-F_1_ mice that was of similar magnitude to non-chimeric WT-F_1_ (B6 x BALB/c) infected mice (Fig 3B). We also detected a small B8_19-26_-specific response in BALB/c→*Batf3*^*-/-*^-F_1_, but the frequency of B8_19-26_-specific CD8^+^ T cells was significantly lower (~12-fold) than in B6→*Batf3*^*-/-*^-F_1_ and WT-F_1_ mice. A small, yet detectable response to F2_26-34_ was also detected in the lungs of B6→*Batf3*^*-/-*^-F_1_-infected mice, but it was ~3 fold and ~8 fold lower in comparison to WT-F_1_- and BALB/c→*Batf3*^*-/-*^-F_1_-infected mice respectively. Thus, these data suggest cross-dressing contributes minimally to priming against these peptide determinants.

It is possible that cross-dressing by H-2K^b^- and H-2L^d^-restricted epitopes other than B8_19-26_ and F2_26-34_, respectively, occurred in infected chimeric mice, so we also performed *ex vivo* stimulations with ΔMHCIi-infected DC2.4 (H-2^b^) and P815 (H-2^d^) cells as these cells present a broad array of naturally derived CPXV peptides (Fig 3B). The frequency of IFN-γ^+^ CD8^+^ T cells upon stimulation with ΔMHCIi-infected DC2.4 cells was significantly lower in BALB/c→*Batf3*^*-/-*^-F_1_ mice in comparison to B6→*Batf3*^*-/-*^-F_1_ and WT-F_1_ mice. Similarly, the frequency of IFN-γ^+^ CD8^+^ T cells upon stimulation with ΔMHCIi-infected P815 cells was significantly lower in B6→*Batf3*^*-/-*^-F_1_ mice in comparison to BALB/c→*Batf3*^*-/-*^-F_1_ and ~10 fold lower in comparison WT-F_1_ mice. The frequency of CD8^+^ T cells that responded to ΔMHCIi-infected P815 cells was also significantly lower in WT-F_1_ in comparison to BALB/c→*Batf3*^*-/-*^-F_1_. This was also seen in F2_26-34_ responses (Fig 3B). These findings may be due to the additional epitope diversity from H-2^b^ as well as H-2^d^ expression in WT-F_1_, which may compromise responses to H-2^d^-restricted epitopes during the primary response. Regardless, these results suggest that cross-dressing from non-hematopoietic cells does not generate a vigorous response during primary CPXV responses.

We next assessed whether cross-dressing plays a role during secondary responses to CPXV infection since cross-dressed APCs are capable of stimulating memory CD8^+^ T cells [9]. However, the secondary CPXV response in the B6→*Batf3*^*-/-*^-F_1_ and BALB/c→*Batf3*^*-/-*^-F_1_ mice were similar to what was observed in the primary CPXV response (Fig 3C). Thus, cross-dressing from non-hematopoietic cells also plays a minor role in activating endogenous memory CD8^+^ T cells following CPXV infection.

To test if cross-dressed MHCI could be contributed by the hematopoietic compartment, we reconstituted lethally irradiated *Batf3*^*-/-*^-F_1_ mice with a 1:1 mixture of BALB/c-Thy1.1 and *Batf3*^*-/-*^-F_1_ BM (S1A Fig). In these mice, cross-presentation should only be carried out by the donor BALB/c-Thy1.1-derived APCs (H-2^d^). In contrast, cells that are of the donor *Batf3*^*-/-*^-F_1_ (H-2^b^ x H-2^d^) origin will lack BATF3^+^ DCs and should not carry out cross-presentation, but may serve as a source of cross-dressing peptide-MHCI complexes. We systemically infected BALB/c-Thy1.1 + *Batf3*^*-/-*^-F_1_→*Batf3*^*-/-*^-F_1_ mice with WT CPXV and found that the H-2^d^-restricted response was successfully reconstituted, whereas the H-2^b^-restricted response was significantly lower than the response in WT-F_1_ mice and was comparable to *Batf3*^*-/-*^-F_1_→*Batf3*^*-/-*^-F_1_ control mice (S1B Fig). These data indicate that APCs cross-dressed from other hematopoietic cells does not efficiently prime CD8^+^ T cell responses in the setting of effective viral MHCI inhibition.

Taken together, these data suggest that antigens are predominantly cross-presented by BATF3^+^ DCs during CPXV infection and that cross-dressing plays a minor role, if at all.

### Cross-presentation of SDEs in the absence of the IDE induces a robust CD8^+^ T cell response that is not affected by viral MHCI inhibition, revealing immunodomination

Insufficient cross-presentation of SDEs may explain the subdominance of other CPXV antigens. To test if cross-presentation of CPXV SDEs alone is capable of inducing a strong CTL response, we mutated the B8_19-26_ epitope anchor residues required for binding to H-2K^b^ peptide-binding groove, postulating that this will prevent the B8_19-26_ epitope from being presented by H-2K^b^. According to the peptide-binding motif of H-2K^b^, the B8_19-26_ epitope contains a primary anchor residue (phenylalanine at position P5) and an auxiliary anchor residue (tyrosine at position P3) [38]. To determine whether mutating the primary anchor residue is sufficient to eliminate binding to H-2K^b^ or if both anchor residues should be mutated, peptide-binding assays were performed using the transporter associated with antigen processing 2 (TAP2)-deficient RMA-S cell line in which addition of peptides capable of binding H-2K^b^ stabilize its expression on the cell surface [39]. Alanine substitution of the primary anchor residue significantly reduced binding of the B8_19-26_ epitope peptide to H-2K^b^ as compared to WT B8, but binding could be increased with increasing concentrations of peptide (S2A Fig). However, alanine substitutions of the primary and auxiliary anchor residues completely abrogated binding of the B8_19-26_ epitope peptide to H-2K^b^, even at higher peptide concentrations. Based on these findings, we introduced both substitutions into the WT and the ΔMHCIi CPXV genomes. The CPXV B8_19-26_ epitope mutants B8Y3AF5A (referred to as ΔB8_19-26_) and a *B8R* deletion mutant (ΔB8R) that we generated did not exhibit defects in viral replication *in vitro* (S2B Fig). Surprisingly, they also did not show attenuated virulence *in vivo*, as measured by weight loss or lethality, as compared to WT CPXV (S2C Fig).

**Fig 4:**
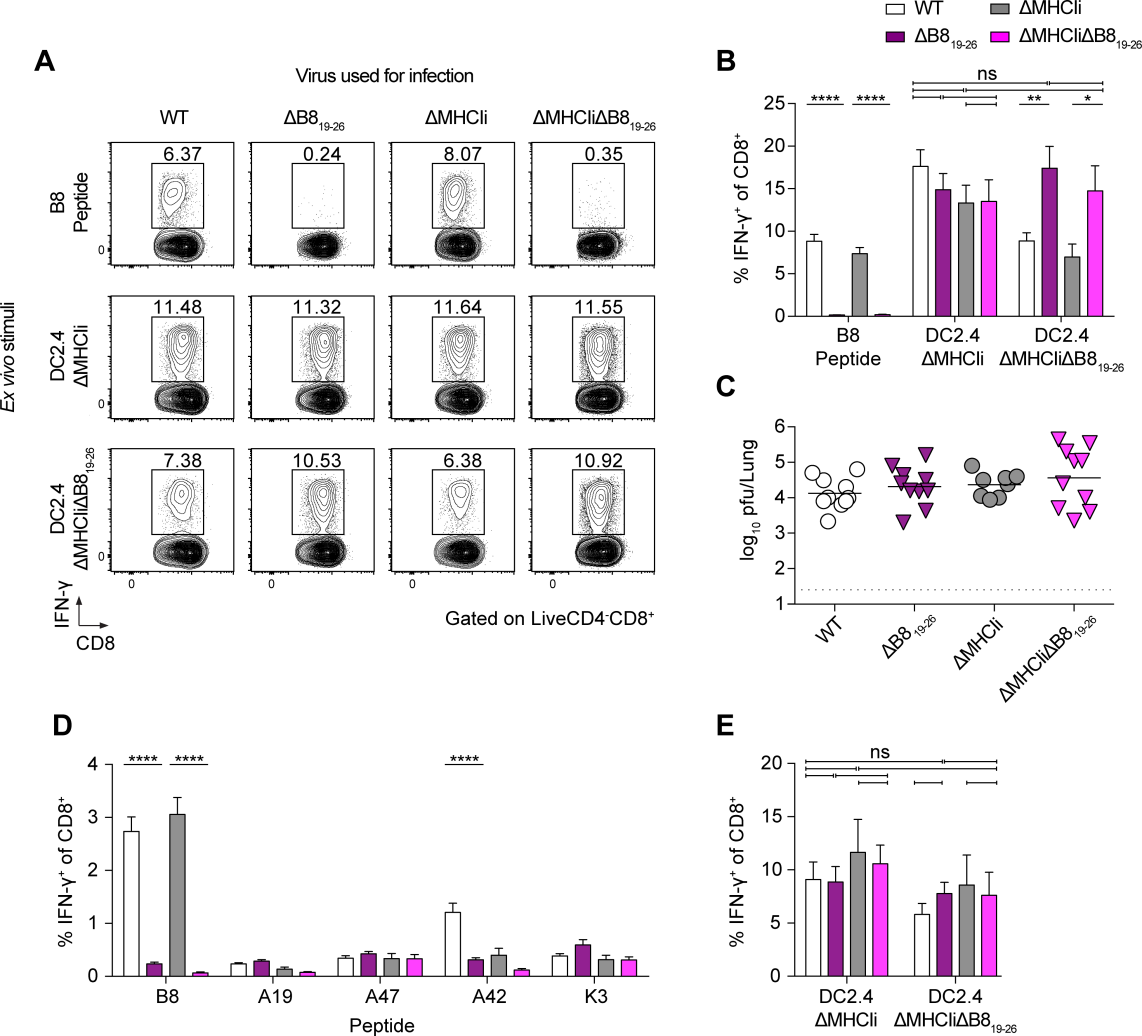
Cryptic subdominant epitopes can compensate for the loss of the CPXV immunodominant epitope-specific CTL response, revealing immunodomination. CTL immunodomination occurs during primary responses against CPXV. (A and B) B6 mice (n = 11–13) were infected i.n with 5 x 10^3^ pfu of WT, ΔB8_19-26_, ΔMHCIi, or ΔMHCIiΔB8_19-26_ and were sacrificed at 8dpi. CD8^+^ T cells in the lungs were restimulated with B8_19-26_ peptide or DC2.4 cells infected with ΔMHCIi or ΔMHCIiΔB8_19-26_. The legend to B indicates the viruses used for infections and the X-axis indicates the stimuli used for *ex vivo* restimulation and ICS. Data are the combined results of five independent experiments. (C) WT and mutant viral strains replicate to similar titers. Viral titers in the lungs of infected B6 mice were determined at 8 dpi by plaque assay. (D and E) Comparable CTL responses against all viral strains tested. B6 mice (n = 6) were infected by i.p. and splenic CD8^+^ T cells were restimulated with peptides (D) or with DC2.4 cells infected with ΔMHCIi or ΔMHCIiΔB8_19-26_ (E). Data are the combined results of four independent experiments.

There was no detectable B8_19-26_ response in ΔB8_19-26_- or ΔMHCIiΔB8_19-26_-infected mice (Fig 4A and 4B, S3A and S3B Fig). However, infections with ΔB8_19-26_ or ΔMHCIi-ΔB8_19-26_ generated a robust SDE response. In contrast, as we previously reported [5], a large proportion of the CPXV-specific CTL response was directed against B8_19-26_ in the lungs of WT- and ΔMHCIi-infected mice. Additionally, there were no significant differences between the overall CTL responses against WT, ΔB8_19-26_, ΔMHCIi, and ΔMHCIiΔB8_19-26_ (Fig 4A and 4B), despite the loss of the B8_19-26_-specific response. Therefore, the CTL response was completely compensated by SDEs in the absence of a B8_19-26_ response.

It was possible that the B8_19-26_ epitope mutation allows CPXV to replicate to higher titers in the lungs of infected mice resulting in higher antigen loads, which could explain the observed compensation. However, the B8_19-26_ epitope mutations did not result in significantly increased viral titers in infected mice (Fig 4C), suggesting that the compensation is unlikely due to increased antigen loads.

Immunodomination and priming by SDEs were also not affected by CPXV-mediated MHCI inhibition since there were no significant difference in the SDE response against ΔB8_19-26_ and ΔMHCIiΔB8_19-26_, as measured by stimulation with ΔMHCIi- (used to estimate total response) or ΔMHCIiΔB8_19-26_- (used to estimate total SDE response) infected DC2.4 cells (Fig 4A and 4B). Additionally, there was no significant difference in the frequency of CD8^+^ T cells that exhibited an effector T cell phenotype in infected mice (S3C and S3D Fig). Considering that the route of infection can alter antigen levels and immunodominance [23], we infected mice by intraperitoneal (i.p.) injections. Compensation by SDEs was also observed during systemic infection (Fig 4D and 4E), suggesting that compensation was not dependent on antigen levels or the route of infection. However, CTL responses against the panel of subdominant epitopes we tested were not significantly increased in the absence of B8_19-26_, suggesting that other unidentified or cryptic subdominant epitopes compensated the CTL response. Interestingly, the response against A42_88-96_ was significantly reduced in the absence of the B8_19-26_-specific response (Fig 4D), suggesting that SDEs were up-ranked in the dominance hierarchy and were now themselves eliciting immunodomination. Furthermore, we found that priming of SDE-specific CD8^+^ T cells was also dependent on BATF3^+^ DCs (S3E Fig). These data suggest that the IDE-specific CTL response suppresses cross-priming of SDE-specific CD8^+^ T cells during primary CPXV infections, indicating immunodomination, but this process was not affected by viral MHCI inhibition.

### SDE-specific CD8^+^ T cell are effective at immunodomination during primary and secondary CPXV infection

**Fig 5:**
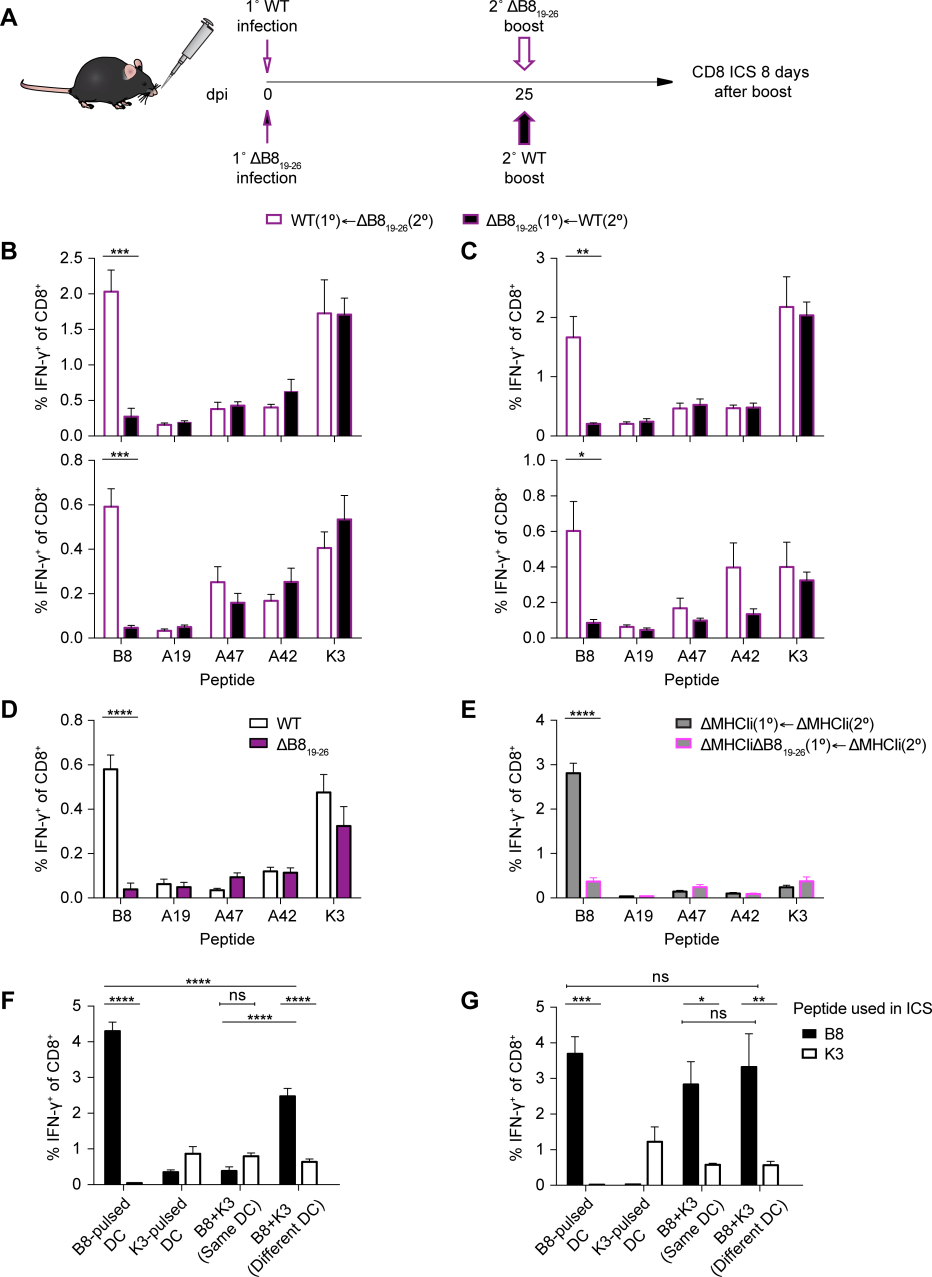
CPXV subdominant epitope-specific memory CTLs immunodominate responses by naïve CD8^+^ T cells. (A) Schematic of i.n. prime/boost experiment. (B and C) Immunodomination of naïve CD8^+^ T cells. B6 mice (n = 5–6) were primed i.n. with 5 x 10^3^ pfu, i.n. boosted at 25 dpi with 5 x 10^3^ pfu (B) or 5 x 10^4^ pfu (C), and sacrificed 8 days after boosting. CD8^+^ T cell responses in the lungs (top) and spleens (bottom) were determined by ICS. Data are the combined results from two independent experiments. (D) Generation of memory CD8^+^ T cells. i.n. primed mice were sacrificed at 25 dpi and memory CD8^+^ T cells were measured in the spleen by ICS. (E) Antibody-independent memory CTL immunodomination. μmT mice were primed by s.s. with 1 x 10^5^ and i.n. boosted with 1 x 10^5^ pfu at 25 dpi. CD8^+^ T cell responses in the spleens were determined 7 days after boost. Data are the combined results from two independent experiments. (F) Memory CTLs cross-compete for peptide-MHCI complexes on APCs. Peptide-pulsed BMDCs were adoptively transferred by tail vein injection into ΔB8_19-26_-primed B6 mice (n = 4) and CD8^+^ T cell responses in the spleen were evaluated by ICS 6 days after transfer. (G) Naïve CD8^+^ T cells do not cross-compete for peptide-MHCI complexes on APCs. Peptide-pulsed BMDCs were transferred into naïve B6 mice and CD8^+^ T cell responses were evaluated by ICS as in the experimental setup of F. Data are representative of two independent experiments.

Memory CD8^+^ T cells also have a capacity for immunodomination and can inhibit naïve CD8^+^ T cell responses [22]. However, this is not the case for VACV since prior priming with individual SDEs does not alter the immunodominance hierarchy following VACV boost in SDE-primed mice [40]. Considering that the priming mechanisms are different during VACV and CPXV infection (Fig 2A), we tested whether CPXV-specific memory CD8^+^ T cells can exert immunodomination. We primed mice with WT CPXV, boosted the mice with a low or high dose of ΔB8_19-26_ at 25 dpi, and assessed the CD8^+^ T cell response in the lungs and spleens 8 days after boosting (Fig 5A). In this group, B8_19-26_-specific memory CD8^+^ T cells should be present pre- and post-boost, but will not undergo expansion following boost with ΔB8_19-26_. As expected, we detected B8_19-26_-specific CD8^+^ T cells in the lungs and spleens of WT CPXV-primed mice after boosting with ΔB8_19-26_ (Fig 5B and 5C) and before boosting (Fig 5D). Additionally, we found that WT and ΔMHCIi infection resulted in a similar relative abundance of B8_19-26_-specific CD8^+^ T cells with a memory phenotype (CD44^+^CD62L^+^KLRG1^-^CD127^+^) at 25 dpi, suggesting that viral MHCI inhibition does not affect memory T cell development (S4 Fig). In a separate group, mice were primed with SDEs by ΔB8_19-26_ infection and boosted with WT CPXV. In this group, we would expect mice to mount a naïve B8_19-26_ response after boosting with WT CPXV only in the absence of memory CD8^+^ T cell immunodomination. However, the naïve B8_19-26_ response was significantly inhibited following boost with both a low and high dose of WT CPXV, suggesting that the SDE-specific memory CD8^+^ T cells immunodominate naïve CD8^+^ T cells. Alternatively, neutralizing antibodies may have reduced the antigen levels and therefore limited the naïve B8_19-26_ response following boost with CPXV.

To assess the potential role of host-protective antibodies, we repeated the above experiments, but this time we depleted CD8^+^ T cells prior to challenging mice with CPXV (S5A and S5B Fig) and then monitored the mice for survival. CPXV-immunized mice that received CD8-depleting or isotype control antibodies survived, whereas naïve mice succumbed to the challenge (S5C Fig). Although this was somewhat expected because CPXV evades CTLs, these results suggest that host-protective antibodies may contribute to protection in the absence of CD8^+^ T cells during secondary exposure to CPXV. We thus repeated the prime and boost experiments and examined immunodomination in μmT mice, which lack mature B cells. Because CPXV evades CTLs *in vivo* and μmT mice should not mount a protective antibody response, it is likely that μmT mice are highly susceptible to WT CPXV infection. To avoid this issue, we infected μmT mice with ΔMHCIi CPXV strains as CTLs can effectively control these viruses in WT mice. We primed μmT mice by skin scarification (s.s.) infection, which resembles human immunizations with VACV. We then boosted the mice at 25 dpi by i.n. administration, and subsequently assessed the CD8^+^ T cell response 7 days after boost. Mice primed with ΔMHCIi resulted in expansion of a B8_19-26_-specific CD8^+^ T cells following i.n. boost with ΔMHCIi (Fig 5E). Mice primed with ΔMHCIiΔB8_19-26_ also mounted a detectable response against B8_19-26_ following i.n. boost with ΔMHCIi, yet this response was significantly reduced by ~9-fold in comparison to mice immunized with ΔMHCIi. Therefore, memory CD8^+^ T cell immunodomination still occurred in the absence of neutralizing antibodies and viral MHCI inhibition, suggesting that immunodomination may be due to T cell interference.

Because memory CD8^+^ T cells are present at higher frequencies than naïve antigen-specific CD8^+^ T cells, it is likely that memory CD8^+^ T cells have a competitive advantage in accessing APC resources [41–43]. For instance, downregulation of MHCI on infected cells may limit the level of antigen presented during CPXV infection, thereby contributing to T cell cross-competition for peptide-MHCI complexes in the secondary response. Indeed, T cell cross-competition for peptide-MHCI complexes during secondary responses has been demonstrated using a heterologous prime-boost strategy [44], but to our knowledge this has only been directly tested between memory and naïve T cells specific for IDEs. To test if SDE-specific memory CD8^+^ T cells can cross-compete with naïve B8_19-26_-specific CD8^+^ T cells, we performed a competition experiment in which we primed mice with ΔB8_19-26_, adoptively transferred peptide-pulsed BM-derived dendritic cells (BMDCs) at 25 dpi, and then assessed the CD8^+^ T cells responses 6 days after transfer. Transfer of B8_19-26_-pulsed BMDCs into ΔB8_19-26_ -primed mice resulted in a robust B8_19-26_ response (Fig 5F). Likewise, transfer of K3_6-15_-pulsed BMDCs resulted in moderate expansion of K3_6-15_-specific memory CD8^+^ T cells. However, when BMDCs that were pulsed with B8_19-26_ and K3_6-15_ at the same time were transferred the B8_19-26_ response was inhibited, further supporting the findings that memory CD8^+^ T cells immunodominate naïve CD8^+^ T cells. Conversely, B8_19-26_-specific CD8^+^ T cells dominated the response when BMDCs pulsed with B8_19-26_ and K3L_6-15_ at the same time were transferred into naïve mice (Fig 5G). If immunodomination is an effect of cross-competition, then providing BMDCs that exclusively present K3_6-15_ and BMDCs that exclusively present B8_19-26_ alone should overcome the effects of immunodomination. When B8_19-26_-pulsed BMDCs were mixed with K3_6-15_-pulsed BMDCs (pulsed separately) and transferred into ΔB8_19-26_-primed mice, the B8_19-26_ response was significantly greater than in mice that received BMDCs pulsed with B8_19-26_ and K3L_6-15_ at the same time, suggesting that cross-competition plays a role in memory CD8^+^ T cell immunodomination.

Interestingly, the B8_19-26_ response in mice that received the 1:1 mixture of K3L_6-15_-pulsed and B8_19-26_-pulsed BMDCs was significantly lower than in mice that only received B8_19-26_-pulsed BMDCs. Therefore, the partial rescue of the B8_19-26_ response when the epitopes were presented on different APCs suggest that additional factors contribute to immunodomination during secondary responses. In contrast to the secondary response, separating the K3_6-15_ and B8_19-26_ epitopes during primary responses had no effect on immunodomination of B8_19-26_-specific CD8^+^ T cells (Fig 5G), suggesting that cross-competition for peptide-MHCI complexes contributes to immunodomination mainly during secondary responses.

**Fig 6:**
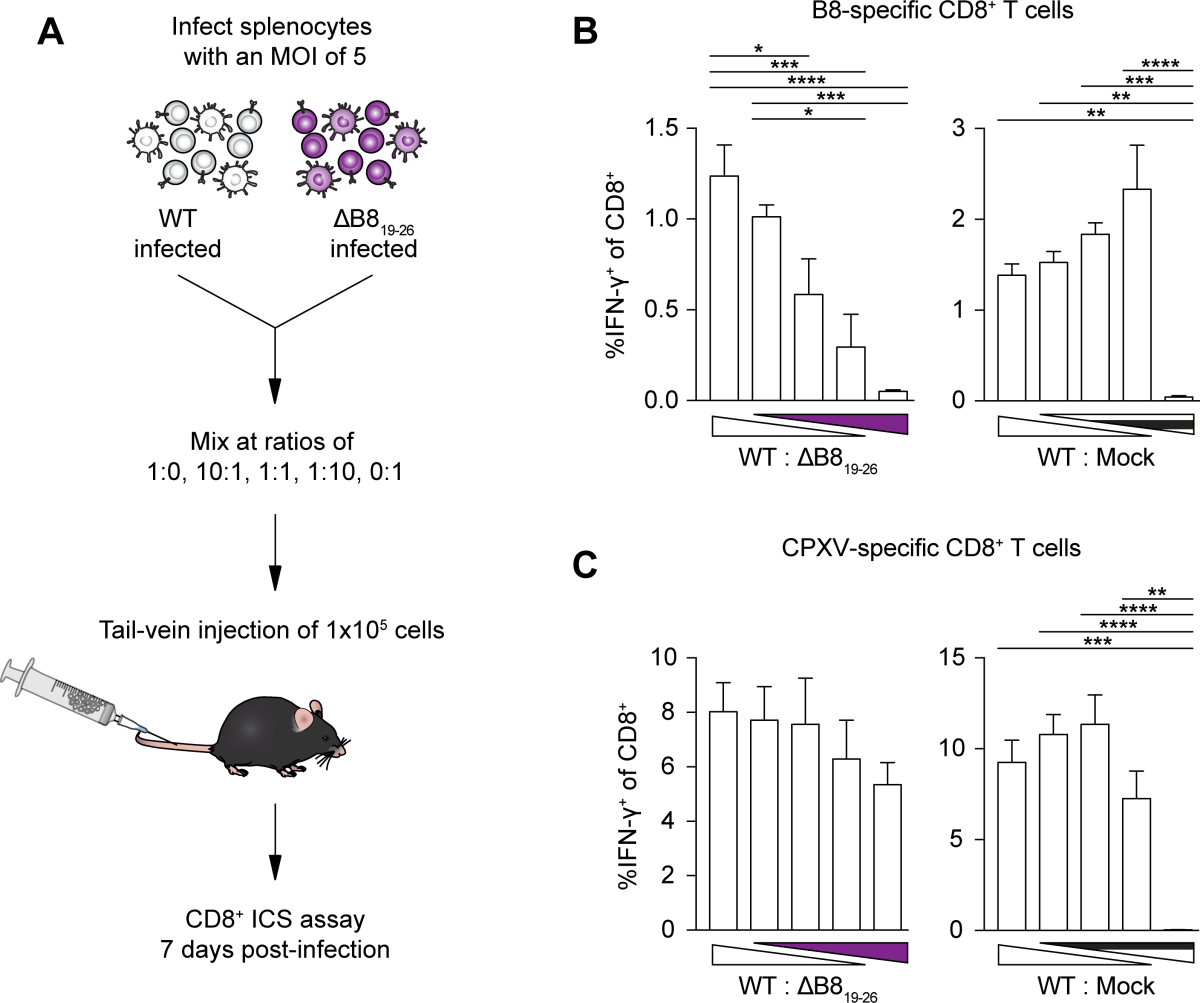
CPXV subdominant epitopes gain dominance when the relative abundance of subdominant antigens is increased during primary responses. (A) Schematic of co-infection experiment. Splenocytes were harvested from B6 mice and infected at an MOI of 5 with WT CPXV and ΔB8_19-26_ separately or mock-infected. At 1 hpi, infected cells were mixed at different ratios and a total of 1 x 10^5^ infected cells were administered into naïve B6 mice (n = 5–6) by tail vein injection. (B and C) A role for antigen levels in CTL immunodomination. Mice were sacrificed at 7 dpi and splenic CD8^+^ T cells were restimulated with B8 peptide (B) or with DC2.4 cells infected with ΔMHCIi (C). Data are the combined results from two independent experiments.

Having demonstrated that SDE-specific memory CD8^+^ T cells have a capacity for immunodomination, we asked if SDE-specific CD8^+^ T cells could exhibit immunodomination during primary responses. We reasoned that modulating the immunodominant and subdominant antigen levels may allow SDE-specific CD8^+^ T cells to immunodominate. To test this, we performed co-infection experiments in which the level of WT and ΔB8_19-26_ input were varied while maintaining the overall viral dose. We first synchronized the infections to limit the variation in the dose by infecting freshly harvested splenocytes with either WT or ΔB8_19-26_ separately. We then mixed WT- and ΔB8_19-26_-infected splenocytes at a ratio of 1:0, 10:1, 1:10, or 0:1, inoculated mice intravenously (i.v.) with a total of 1 x 10^5^ infected cells, and assessed the CTL response at 7 dpi (Fig 6A). A graded B8_19-26_ response was observed with the concurrent increase of ΔB8_19-26_ input and decrease of WT input (Fig 6B), while the overall response as determined by stimulation with ΔMHCIi-infected DC2.4 cells remained roughly equal (Fig 6C). These data suggest that SDE-specific CTLs are capable of immunodominating the primary response when the relative abundance of subdominant antigens is increased, even in the presence of the IDE. To confirm that the graded response was not simply due to reduced WT input, we repeated the co-infection experiment using mixtures of WT- and mock-infected splenocytes. Injecting the varying mixtures of WT- and mock-infected splenocytes did not result in a gradation of the B8_19-26_ response (Fig 6B), suggesting that the observed graded B8_19-26_ response was dependent on the subdominant antigen levels.

### SDE-primed CD8^+^ T cells control lethal CPXV infection in the absence of the CPXV MHCI inhibitors

**Fig 7:**
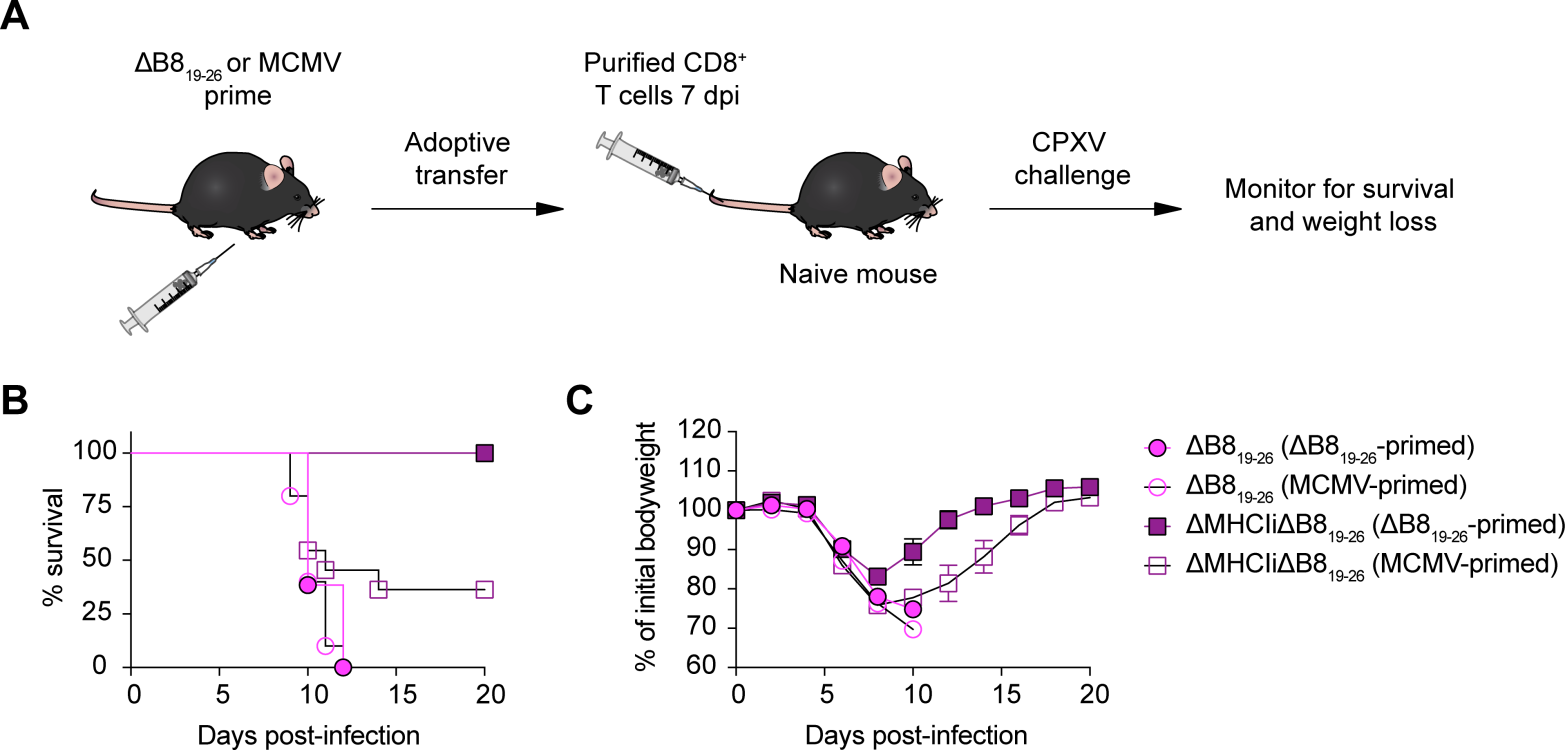
Subdominant epitope-specific CTL responses protect against CPXV infection. (A) Schematic of adoptive transfer experiment. B6 mice were primed with ΔB8_19-26_ or MCMV by subcutaneous (s.c.) or i.p. routes, respectively. At 7 dpi, splenic CD8^+^ T cells were isolated by positive selection and adoptively transferred into naive B6 mice (n = 11–13) by tail vein injection. After ~1 day, mice were infected by i.n. inoculation with ΔB8_19-26_ or ΔMHCIiΔB8_19-26_ and monitored for survival (B) and weight loss (C). Data are the combined results from two independent experiments.

Thus far, our results indicate that CPXV-mediated MHCI inhibition does not affect priming of CD8^+^ T cells by SDEs. However, we wondered whether SDE-specific CTL responses could provide protection against CPXV infection *in vivo*. To examine the physiological relevance of SDEs in protecting against CPXV infection, we performed adoptive transfer experiments with CTLs primed with ΔB8_19-26_ or MCMV as a control for antigen specificity (Fig 7A). Mice that received primed CTLs were then challenged by i.n. administration with a lethal dose of ΔB8_19-26_ or ΔMHCIiΔB8_19-26_. The majority of mice that received MCMV-primed CTLs died following infection with ΔB8_19-26_ or ΔMHCIiΔB8_19-26_ (Fig 7B and 7C). All mice that received ΔB8_19-26_-primed CTLs also died after challenge with ΔB8_19-26_, whereas all mice challenged with ΔMHCIiΔB8_19-26_ survived. Therefore, CTLs primed by SDEs are capable of recognizing and controlling CPXV only in the absence of CPXV-mediated MHCI inhibition, which is consistent with our previous findings regarding WT CPXV exposure that is dominated by the B8_19-26_ response [4,5].

## Discussion

Here we demonstrate that the secretion of an immunodominant CPXV antigen does not affect immunodominance or cross-priming by the IDE. Intriguingly, we found that the IDE and SDEs are differentially presented by APCs during infection with CPXV and VACV, despite being closely related genetically. We also show that CD8^+^ T cell immunodomination is not affected by viral MHCI inhibition and can be elicited by SDEs during primary and secondary responses against CPXV infection. Additionally, we show that SDEs alone are entirely capable of generating protective CTL responses, which is dependent on cross-priming by BATF3^+^ DCs.

Cross-priming of CD8^+^ T cells is important for inducing antiviral CTL responses, especially in settings where direct-presentation is not possible (e.g., APCs are not susceptible to infection) or is evaded (e.g., impairing maturation of infected-APCs or inhibiting MHCI presentation). Consistent with this notion, herein we showed that the induction of antiviral CTL responses is dependent on cross-presentation in the presence of CPXV-mediated MHCI inhibition. Priming of CD8^+^ T cell in the absence of viral MHCI inhibition during CPXV infection was also dependent on cross-presenting BATF3^+^ DCs, albeit to a less extent. While we have not ruled out the possibility that ΔMHCIi-infected BATF3^+^ DCs prime CTL precursors by direct presentation, CPXV-infected DCs have reduced expression of costimulatory molecules involved in T cell activation [45,46], suggesting that direct-presentation may be limited even in the absence of CPXV012 and CPXV203. Nonetheless, there are clearly factors other than MHCI inhibition that skew priming of T cells towards cross-priming and further study of CPXV ORFs in the context of ΔMHCIi provides an excellent opportunity to investigate such factors. In this study, we provide evidence that cross-priming is the main mechanism driving CPXV-specific CTL responses.

Our studies also indicate that cross-dressing plays no significant role in the T cell response to CPXV infections *in vivo*. Cross-dressing has been proposed as a mechanism by which APCs can rapidly acquire peptide epitopes for presentation to CTL precursors, thereby eliminating the time spent for antigen processing [9,10]. In support of this, DCs can be cross-dressed *in vitro* by peptide-MHCI complexes from epithelial cells [47], which are commonly targeted by viruses and thus may serve as a common source of preformed viral peptide-MHCI. Moreover, peptide-MHCI from parenchymal cells cross-dressed DCs in vesicular stomatitis virus (VSV)-infected mice and the cross-dressed DCs induced proliferation of memory CD8^+^ T cells, but not naïve T cells. However, priming of naïve antigen-specific CD8^+^ T cells by cross-dressed DCs can occur, as demonstrated using DNA vaccination and transfer of adenovirus infected DCs [10,11]. In contrast to these studies, we found that cross-dressing does not efficiently prime or drive expansion of endogenous antigen-specific naïve and memory CD8^+^ T cells during CPXV infection.

While previous reports on cross-dressing provide compelling evidence that cross-dressing occurs *in vivo*, the transfer of TCR tg T cells in these studies may have resulted in non-physiological induction of CD8^+^ T cells by cross-dressed DCs. Additionally, cross-dressing in these experimental settings may have been promoted due to a potential generation of supraphysiological levels of peptide-MHCI by DNA vaccination or by transfer of adenovirus infected DCs. These factors may explain the difference between previous studies and our results using CPXV infection. Because CPXV encodes an extensive arsenal of immunomodulatory proteins, the possibility that CPXV directly or indirectly inhibits cross-dressing may also explain these conflicting results. For example, downregulation of MHCI cell surface expression by CPXV012 and CPXV203 may prevent transfer of peptide-loaded MHCI molecules by trogocytosis, a process in which intercellular exchange of intact membranes occurs during the formation of an immunological synapse [48–51]. If trogocytosis is required for cross-dressing of APCs *in vivo*, as been demonstrated *in vitro* [9], then cross-dressing dependent T cell responses are expected to be abrogated during CPXV infection. Ultimately, our results suggest that antigens are acquired from necrotic/apoptotic bodies or secreted viral proteins found in the extracellular milieu and are then predominantly cross-presented during CPXV infection.

Cross-presentation of peptide epitopes may also be influenced by the nature of the antigens and can affect the extent of CD8^+^ T cell immunodominance [52–54]. For instance, the secreted immunodominant antigens of *M*. *tuberculosis* are likely processed through the cross-presentation pathway [55,56] and eliminating bacterial secretion prevents priming of IDE-specific CD8^+^ T cells during *M*. *tuberculosis* infection [31]. Priming of naïve CD8^+^ T cells against cell-associated subdominant SV40 large tumor antigen (T Ag) epitope V is also dependent on cross-presentation, but the response against the V epitope is limited because it is inefficiently cross-presented relative to the T Ag IDE [54]. Our findings suggest that cross-presented CPXV IDEs can be derived from cell-associated antigen since ablating B8 secretion did not negatively affect cross-priming dependent induction of B8_19-26_-specific CD8^+^ T cells. Moreover, cell-associated B8 elicited a greater B8_19-26_-specific CD8^+^ T cell response in comparison to secreted soluble B8, which is consistent with the preferential *in vivo* cross-presentation previously reported for cell-associated antigens [33,34]. However, the underlying mechanisms of immunodominance are complex and are often context dependent as we found that secretion of CPXV B8 is not required for immunodominance and that cross-presentation of CPXV SDEs in the absence of the immunodominant B8_19-26_ epitope stimulated a robust CTL response. The fact that the CTL response to SDEs compensated for the absence of B8_19-26_ suggests that the SDE response is suppressed by the B8_19-26_ response, supporting the concept of immunodomination.

In many cases immunodomination occurs as a consequence of T cell competition for limiting APC resources [41,43,57,58]. For instance, competition for peptide-MHCI complexes on APCs during primary CTL responses can occur as a result of antigen abundance [59]. In support of this, we showed that concurrently increasing subdominant antigen levels and reducing immunodominant antigen levels allow SDEs to gain dominance during the primary response to CPXV infection. Similarly, modulating the antigen abundance through different methods during influenza A virus and VACV infection has been shown to influence immunodomination [23,60]. In certain models, immunodomination can be overcome when APCs present different epitopes separately [42,51,58], indicating that CD8^+^ T cells of different specificities can cross-compete for peptide-MHCI complexes on APCs. This has been convincingly demonstrated in models where immunodomination occurs when APCs co-present model antigen epitopes. However, epitope co-presentation by APCs does not always influence immunodomination, as we have shown here for primary responses, and the role of cross-competition in inducing antiviral CTL responses is controversial [61].

We found that cross-competition for peptide-MHCI complexes is relevant and that immunodomination occurs during secondary responses as a consequence. Alternatively, the suppressed B8_19-26_ response in our cross-competition experiments may have resulted from K3_6-15_-specific memory CD8^+^ T cells killing the BMDCs that were pulsed with B8_19-26_ and K3L_6-15_ at the same time. Nevertheless, we observed partial rescue of the B8_19-26_ response when the epitopes were separated on BMDCs. This partial rescue may be due to peptide exchange between BMDCs that were pulsed separately and adoptively transferred as a mix, which would subsequently result in epitope co-presentation and K3_6-15_-specific memory CD8^+^ T cell immunodomination. However, additional factors that we did not test such as cross-competition for growth factors, antigen-specific T cell precursor frequencies, or TCR avidity [62] likely contribute to the memory T cell immunodomination as well.

Remarkably, immunodomination during the secondary response against CPXV was exerted by SDE-specific memory CD8^+^ T cell. The capacity for SDE-specific memory CD8^+^ T cells to inhibit the response to an IDE has been shown with influenza virus [22], but prior priming with SDE peptides did not result in memory CD8^+^ T cell immunodomination using VACV, as shown by Wang et al [40]. Here in our study, memory CD8^+^ T cell immunodomination was clearly evident when SDE-primed mice were challenged with WT CPXV, whereby the naïve B8_19-26_-specific CD8^+^ T cell response was suppressed. Moreover, memory CD8^+^ T cell immunodomination was not affected by MHCI inhibition. However, mice were primed by CPXV infection (in this study) as opposed to individual SDE peptides (as done by Wang et al). These experimental differences suggest that the priming stimulus and the breadth of the primary response influences immunodomination during secondary responses against poxviruses.

Taken together, our findings highlight the need to consider the effects of pre-existing immunity on the outcome of secondary responses and vaccinations. An advantage to using VACV-based vaccines is that in addition to providing protection against heterologous pathogens, the native vector epitopes (both IDEs and SDEs) can provide cross-protection against related orthopoxviruses, as supported by our findings here and previous reports [63–66]. However, as a consequence of pre-existing immunity, memory CD8^+^ T cell immunodomination may limit the target antigen response following immunization with VACV-based vaccines, in turn resulting in non-efficacious vaccinations. For example, native VACV epitopes can mask responses against target antigens expressed by VACV vaccine vectors [19]. Nevertheless, our results support the ongoing evaluation for poxviruses as promising vaccine vectors, and stress the necessity to develop novel vaccination strategies.

## Materials and methods

### Cell lines, mice and viruses

Cell lines HeLa, Vero, and P815 were obtained from the American Type Culture Collection (ATCC). DC2.4 cells were a kind gift from Dr. Kenneth Rock, University of Massachusetts Medical School. HeLa, Vero, DC2.4, and P815 cells were cultured respectively in Dulbecco’s Modified Eagle Medium (DMEM), Minimum Essential Medium (MEM) or RPMI supplemented with 10% FBS (Mediatech), 100 U/ml penicillin, 100 g/ml streptomycin, 1mM sodium pyruvate, and non-essential amino acids (Gibco). VACV-WR was obtained from the ATCC. MCMV Smith strain was a gift from Dr. Herbert Virgin, Washington University. CPXV BAC pBR mini-F construct was kindly provided by Dr. Karsten Tischer, Free University of Berlin. Mutant viruses were generated by *en passant* mutagenesis [67] using primers listed in S1 Table. Gene fragments were synthesized (Integrated DNA Technologies) and assembled using Gibson Assembly (New England BioLabs) for cloning of the B8-mCherry fusion contructs (S2 Table). Infectious BAC-derived viruses (S3 Table) were reconstituted using a slightly modified method previously described by Xu *et al* [68]. In brief, ~8x10^5^ Vero cells seeded in 6-well plates were infected with fowlpox virus (FWPV) at an MOI of 1. Transfection of FWPV-infected Vero cells was carried out 1 hour post-infection (hpi) with 4 μg of BAC DNA and 5 μL of Lipofectamine 2000 transfection reagent (ThermoFisher Scientific) according to the manufacturer’s instruction. Serial dilutions of reconstituted infectious virus were passaged up to four times on Vero cells in order to remove the mini-F vector sequence. Wells harbouring single GFP-negative plaque were isolated and used for preparing virus stocks as previously described [17]. C57BL/6Ncr mice were purchased from the National Cancer Institute. B6.129S2-Ighm^tm1Cgn^/J mice were purchased from the Jackson Laboratory. *Batf3*^*-/-*^ mice crossed to the C57BL/6 and BALB/c background were kindly provided by Dr. Kenneth Murphy, Washington University. Growth curves were performed on Vero cells. Supernatant and cells were harvested at 12, 24, 28, and 72 hpi and viral titers were determined by plaque assay using Vero cells.

### Peptide binding assay

TAP2-deficient RMA-S (H-2^b^) cells were cultured overnight at 28°C in 5% CO_2_ to accumulate peptide-receptive MHCI molecules at the cell surface. Peptides were then added at various concentrations and the cells were transferred to 37°C. After 6 h of incubation at 37°C, cells were harvested and washed twice in PBS. H-2K^b^ cell surface expression was then measured by flow cytometry.

### Western blot

1 x 10^6^ HeLa cells were infected at a MOI of 5. Cells and supernant were collected at 4 hpi and were lysed on ice for 5 min in RIPA lysis buffer supplemented with 1x Halt protease and phosphatase inhibitor cocktail. Cells were further processed for subcellular fractionation using a Subcellular Protein Fractionation Kit (ThermoFisher). Samples were mixed with Laemmli sample buffer (Bio-Rad), incubated at 95°C for 5 minutes, separated by SDS-PAGE, and transferred to PVDF membranes. Immunoblotting was performed using rabbit polyclonal anti-mCherry and rabbit monoclonal anti-EGFR (Abcam) followed by horseradish peroxidase (HRP)-conjugated goat anti-rabbit IgG (Cell Signalling).

### Generation of bone marrow chimeras

6 weeks of age *Batf3*^*-/-*^*-F*_*1*_ (H2^b^xH2^d^) mice were depleted of NK cells by i.p. administration of 100 μg of PK136 antibody. Two days later, the mice were lethally irradiated with 950 rads and were reconstituted with 1x10^7^ T cell depleted C57BL/6, BALB/c, or a 1:1 mixture of BALB/c-Thy1.1 and *Batf3*^*-/-*^*-F*_*1*_ BM cells. BM chimeras were treated with antibiotics for 4 weeks and were allowed to reconstitute for 8 weeks before use.

### Generation of bone marrow-derived DCs and immunization

BMDCs were generated by culturing BM cells in the presence of 20 ng/mL GM-CSF and IL-4 (PeproTech) for 8 days, as previously described [69]. LPS (150ng/nL) was then added and the cells were allowed to mature overnight. The cells were then pulsed with peptide (1g/mL, 45 min). Cells were washed extensively in PBS and a total of 2.5 x 10^5^ DCs was injected i.v. into recipient mice.

### Mouse infection and CD8^+^ T cell adoptive transfer

Mice were age- and sex-matched for each experiment and used at 8–10 weeks of age. Mice were infected as previously described for i.n. and s.s. infections [5]. For s.s. infections, fur was trimmed with clippers, then a thin layer of Vaseline was applied over the trimmed region and the remaining fur was shaved over with a double-edge razor blade one day before infection. Mice infected by i.p. or s.c. administration were injected with a volume of 100μL or 200μL of virus inoculum per mouse, respectively. For co-infection experiments, splenocytes isolated from B6 mice were infected at an MOI of 5, harvested 1 hpi, and washed three times with PBS. 1 x 10^5^ infected cells in 200 μL of PBS were transferred intravenously into naïve B6 mice. For the CPXV SDE protection experiment, CD8^+^ T cells from splenocytes of B6 mice that had been infected 7 days earlier with WT CPXV or MCMV were isolated by positive selection using anti CD8a MicroBeads (Miltenyi Biotec). 3 x 10^6^ CD8^+^ T cells were transferred intravenously into naïve B6 mice. Mice were infected approximately 24 h after transfer.

### Flow cytometry, IFN-γ production assays, and antibodies

Single-cell suspensions from the lungs and spleens were prepared at the indicated days post-infection as previously described [5]. 1x10^6^ cells were seeded in 96-well plates and were re-stimulated with peptides or with 1x10^5^ DC2.4 cells that had been infected for 4 h with ΔMHCI-i or ΔMHCI-iΔB8 CPXV (MOI 5). Cells were incubated at 37°C, 5% CO_2_. After 1 h at 37°C, GolgiPlug (BD Biosciences) was added to each well. Three hours later, cells were stained on ice with Fixable Viability Dye eFlour 506 (eBioscience) before staining of cell surfaces for the indicated surface markers. Cells were then fixed/permeabilized and stained for IFN-γ. Background levels were determined using cells from uninfected mice, which usually ranged between 0.01–0.05%, and were subtracted from the values presented. For intracellular staining of GzmB and tetramer staining, cells were stained *ex vivo* without stimulation and without incubation with GolgiPlug. H-2K^b^-TSYKFESV tetramers were produced in the Immunomonitoring Laboratory within the Center for Human Immunology and Immunotherapy Programs (Washington University). The following monoclonal antibodies were obtained from ThermoFisher, BD Biosciences or eBioscience: H-2K^b^ (AF6-88.5), CD3 (145-2C11), CD8α (53–6.7), CD8β (eBioH35-17.2), CD4 (RM4-5), CD44 (IM7), CD62L (MEL-14), GzmB (GB12), KLRG1 (2F1), CD127 (A7R34) and IFN-γ (XMG1.2).

### Statistics

The data are shown as mean ± SEM and were analysed with an unpaired Student *t* test or one-way ANOVA followed by Tukey posttest comparison using Prism GraphPad software, asterisks indicate statistical significance and the *p* values are denoted as *p<0.05, **p<0.01, ***p<0.001.

### Ethics statement

Mouse studies were approved by the Animal Studies Committee at Washington University, protocol # A-3381-01, and adhere to the Institutional Animal Care and Use Committee guidelines.

## Supporting information

S1 Fig Cross-dressing by hematopoietic cells does not induce CTL responses during CPXV infection. (A) Schematic of bone marrow chimera cross-dressing experiment.
(B) Hematopoietic cells do not contribute to CTL-priming *via* cross-dressing of APCs. Lethally irradiated *Batf3*^*-/-*^-F_1_ mice (n = 8) reconstituted with a 1:1 mixture of BALB/c-Thy1.1 and *Batf3*^*-/-*^-*F*_*1*_ bone marrow cells were infected i.p. with 1 x 10^5^ pfu WT CPXV and CD8^+^ T cell responses in the spleen were assessed as in the experimental setup in Fig 3. n = 4 WT-F_1_ CPXV-infected mice. The data are the combined results of three independent experiments.
(TIF)

S2 Fig Mutating the CPXV immunodominant CD8^+^ T cell epitope anchor residues alters peptide binding affinity to MHCI H-2K^b^, but does not affect CPXV replication and virulence. Peptide binding assays were performed using RMA-S cells. (A) Peptide anchor residues are critical for H-2K^b^ binding. Cell surface staining of H-2K^b^ after incubation with peptide (black) or without peptide (red) are shown; isotype control staining is shown in grey. Data are representative of three independent experiments. (B) B8 mutations do not affect viral kinetics *in vitro*. Vero cells were infected at an MOI of 0.01 for multi-step growth curves. Data are the combined results of three independent experiments performed in duplicates. (C) B8 mutations do not affect viral pathogenesis *in vivo*. B6 mice (n = 5–9) were infected i.n. with 4 x 10^4^ pfu of the indicated viruses and monitored for survival and weight loss.
(TIF)

S3 Fig Cross-priming of cryptic subdominant epitopes can compensate for the loss of the CPXV immunodominant epitope-specific CTL response. Peptide anchor residues are critical for inducing B8_19-26_-specific CTL responses. (A and B) B6 mice (n = 10) were infected i.n with 5 x 10^3^ pfu WT, ΔB8_19-26_, ΔMHCIi, or ΔMHCIiΔB8_19-26_ and were sacrificed at 8dpi. The B8_19-26_-specific CTL response in the spleen was evaluated by tetramer staining. Data are the combined results from two independent experiments. (C and D) Comparable CTL responses against all viral strains. B6 mice (n = 5) were infected and sacrificed at 8 dpi as in experimental setup of A and B. Cell surface expression of CD62L, CD44 and intracellular GzmB was determined for CD8^+^ T cells in the lungs. Data are representative of three independent experiments. (E) BATF3^+^ DCs cross-prime SDE-specific CTL precursors. B6 or *Batf3*^*-/-*^ mice (n = 7) were infected i.p. with 1 x 10^5^ pfu ΔB8_19-26_ and CD8^+^ T cell responses in the spleen were measured by *ex vivo* restimulation with ΔMHCIiΔB8_19-26_-infected DC2.4 cells. ICS was performed at 8 dpi. Data are the combined results from two independent experiments.
(TIF)

S4 Fig Viral MHCI inhibition does not affect the generation of memory CD8^+^ T cells. (A and B) Generation of memory CD8^+^ T cells following CPXV infection. B6 mice (n = 7) were primed i.n. with 5 x 10^3^ pfu WT or ΔMHCIi and were sacrificed at 25 dpi. Cell surface expression of memory T cell markers (CD62L, CD44, KLRG1, and CD127) was determined for TET^+^CD8^+^ T cells in the spleen. Data are the combined results from two independent experiments.
(TIF)

S5 Fig CPXV-immunized mice survive lethal challenge in the absence of memory CD8^+^ T cells. (A) Schematic of immunization and challenge experiment. B6 mice (n = 6–7) were primed i.n. with 5 x 10^3^ pfu of CPXV and lethally challenged at 25 dpi. Anti-CD8α or isotype control antibodies were administered at the indicated times. (B) Complete depletion of CD8^+^ T cells. The efficiency of antibody-mediated CD8 depletion was determined one day after the first administration of antibodies. (C) CPXV immunized mice generate protective antibody responses. Challenged mice were monitored for survival and weight loss.
(TIF)

S1 Table Primer sequences. (TIF)

S2 Table Synthesized gene fragments. (TIF)

S3 Table Viruses used in this study. (TIF)
