# Supplementary figures and images for: Cross-priming induces immunodomination in the presence of viral MHC class I inhibition
Source: PLoS Pathog. 2018 Feb 14;14(2):e1006883. doi: 10.1371/journal.ppat.1006883 (PMC5812664; doi:10.1371/journal.ppat.1006883)

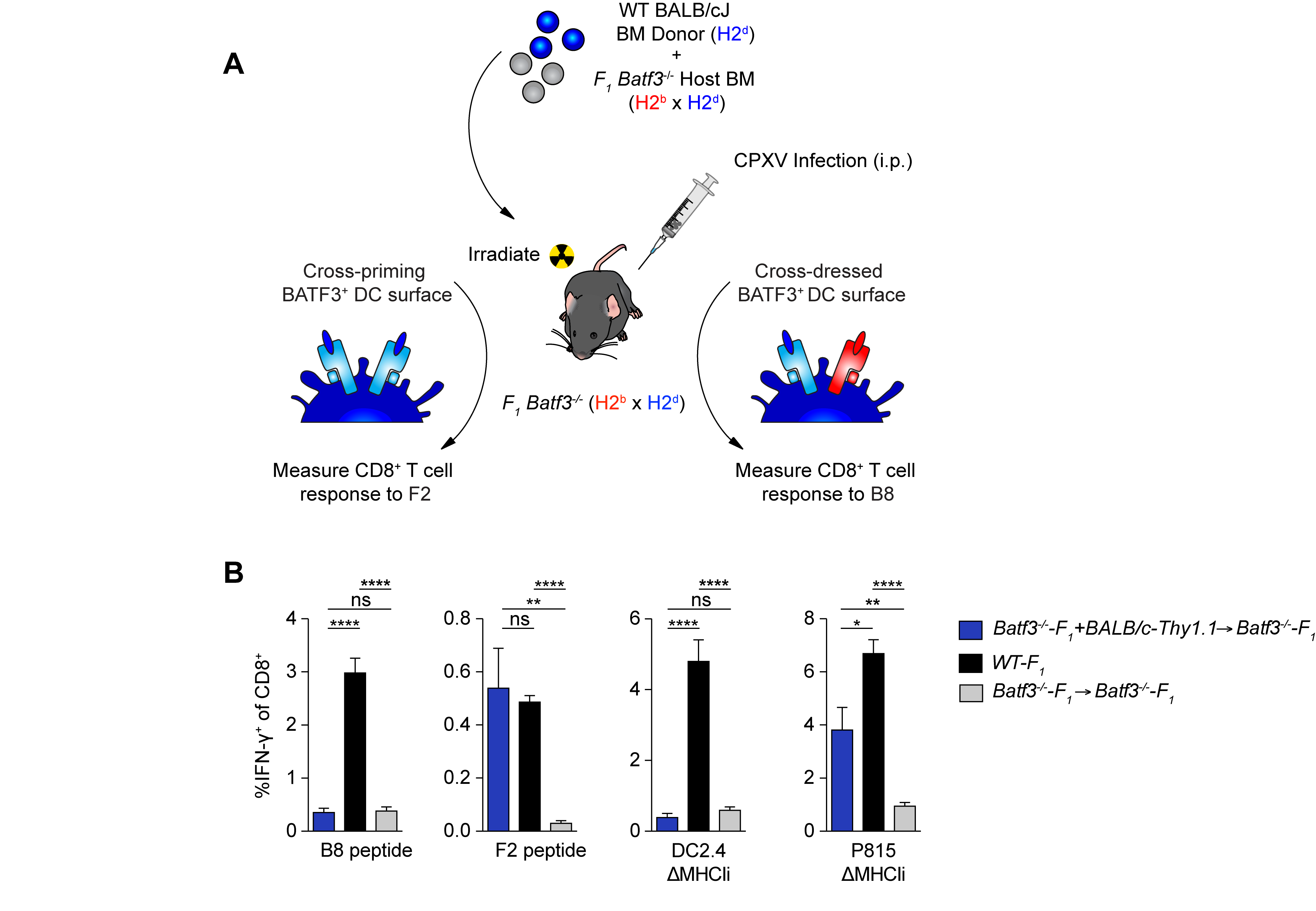

Supplement: S1 Fig — (A) Schematic of bone marrow chimera cross-dressing experiment. (B) Hematopoietic cells do not contribute to CTL-priming via cross-dressing of APCs. Lethally irradiated Batf3-/--F1 mice (n = 8) reconstituted with a 1:1 mixture of BALB/c-Thy1.1 and Batf3-/--F1 bone marrow cells were infected i.p. with 1 x 10^5^ pfu WT CPXV and CD8+ T cell responses in the spleen were assessed as in the experimental setup in Fig 3. n = 4 WT-F1 CPXV-infected mice. The data are the combined results of three independent experiments. (TIF) [file ppat.1006883.s001.tif]

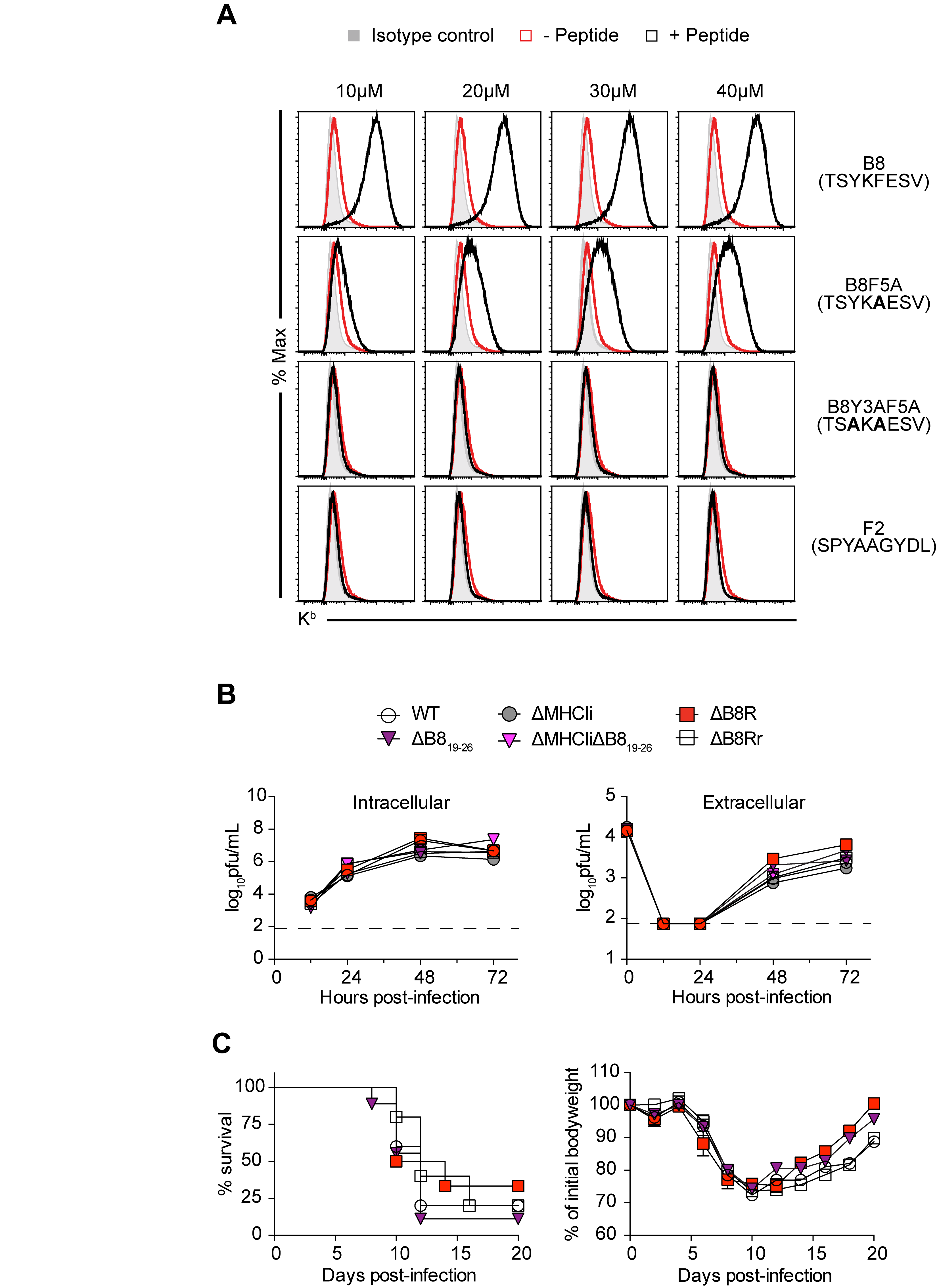

Supplement: S2 Fig — Peptide binding assays were performed using RMA-S cells. (A) Peptide anchor residues are critical for H-2Kb binding. Cell surface staining of H-2Kb after incubation with peptide (black) or without peptide (red) are shown; isotype control staining is shown in grey. Data are representative of three independent experiments. (B) B8 mutations do not affect viral kinetics in vitro. Vero cells were infected at an MOI of 0.01 for multi-step growth curves. Data are the combined results of three independent experiments performed in duplicates. (C) B8 mutations do not affect viral pathogenesis in vivo. B6 mice (n = 5–9) were infected i.n. with 4 x 10^4^ pfu of the indicated viruses and monitored for survival and weight loss. (TIF) [file ppat.1006883.s002.tif]

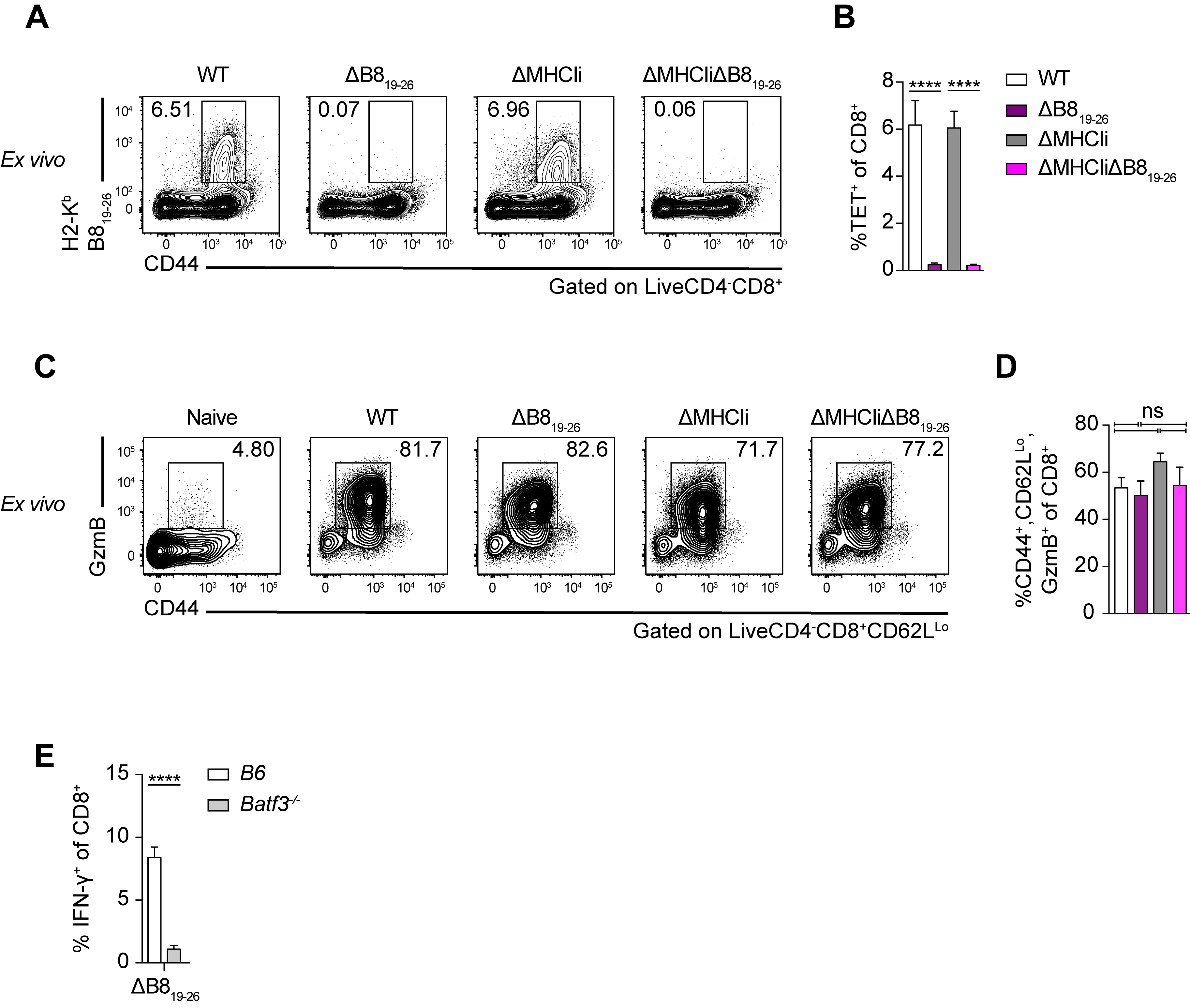

Supplement: S3 Fig — Peptide anchor residues are critical for inducing B819-26-specific CTL responses. (A and B) B6 mice (n = 10) were infected i.n with 5 x 10^3^ pfu WT, ΔB819-26, ΔMHCIi, or ΔMHCIiΔB819-26 and were sacrificed at 8dpi. The B819-26-specific CTL response in the spleen was evaluated by tetramer staining. Data are the combined results from two independent experiments. (C and D) Comparable CTL responses against all viral strains. B6 mice (n = 5) were infected and sacrificed at 8 dpi as in experimental setup of A and B. Cell surface expression of CD62L, CD44 and intracellular GzmB was determined for CD8+ T cells in the lungs. Data are representative of three independent experiments. (E) BATF3+ DCs cross-prime SDE-specific CTL precursors. B6 or Batf3-/- mice (n = 7) were infected i.p. with 1 x 10^5^ pfu ΔB819-26 and CD8+ T cell responses in the spleen were measured by ex vivo restimulation with ΔMHCIiΔB819-26-infected DC2.4 cells. ICS was performed at 8 dpi. Data are the combined results from two independent experiments. (TIF) [file ppat.1006883.s003.tif]

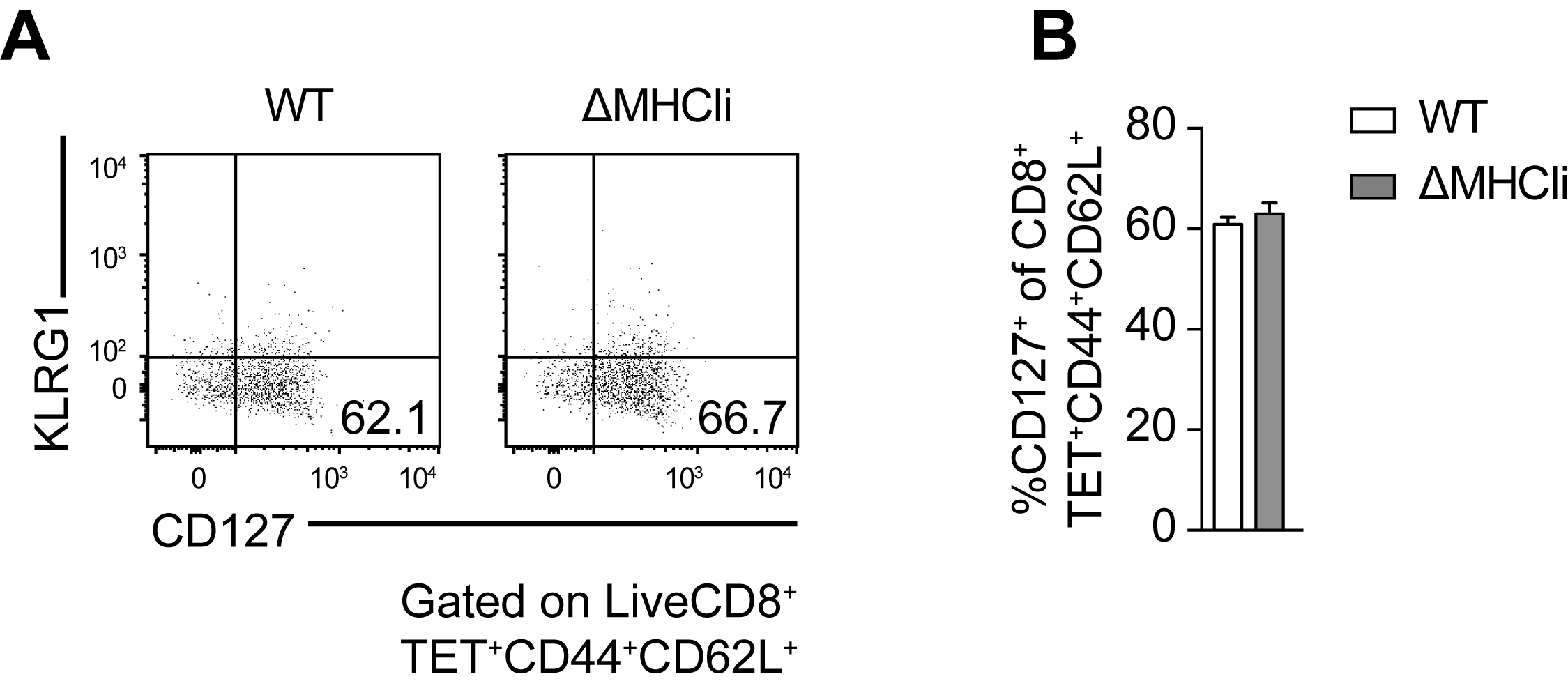

Supplement: S4 Fig — (A and B) Generation of memory CD8+ T cells following CPXV infection. B6 mice (n = 7) were primed i.n. with 5 x 10^3^ pfu WT or ΔMHCIi and were sacrificed at 25 dpi. Cell surface expression of memory T cell markers (CD62L, CD44, KLRG1, and CD127) was determined for TET+CD8+ T cells in the spleen. Data are the combined results from two independent experiments. (TIF) [file ppat.1006883.s004.tif]

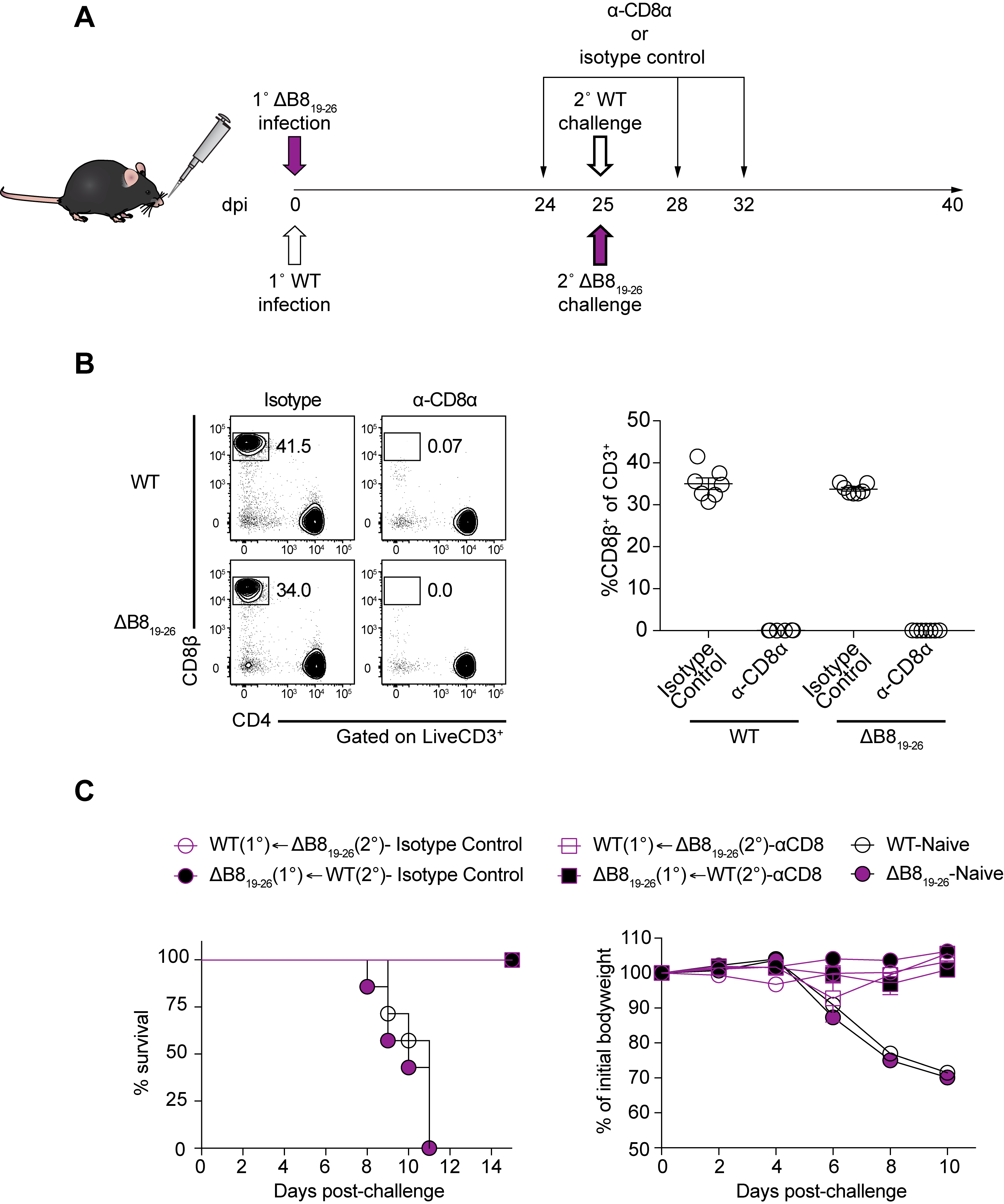

Supplement: S5 Fig — (A) Schematic of immunization and challenge experiment. B6 mice (n = 6–7) were primed i.n. with 5 x 10^3^ pfu of CPXV and lethally challenged at 25 dpi. Anti-CD8α or isotype control antibodies were administered at the indicated times. (B) Complete depletion of CD8+ T cells. The efficiency of antibody-mediated CD8 depletion was determined one day after the first administration of antibodies. (C) CPXV immunized mice generate protective antibody responses. Challenged mice were monitored for survival and weight loss. (TIF) [file ppat.1006883.s005.tif]

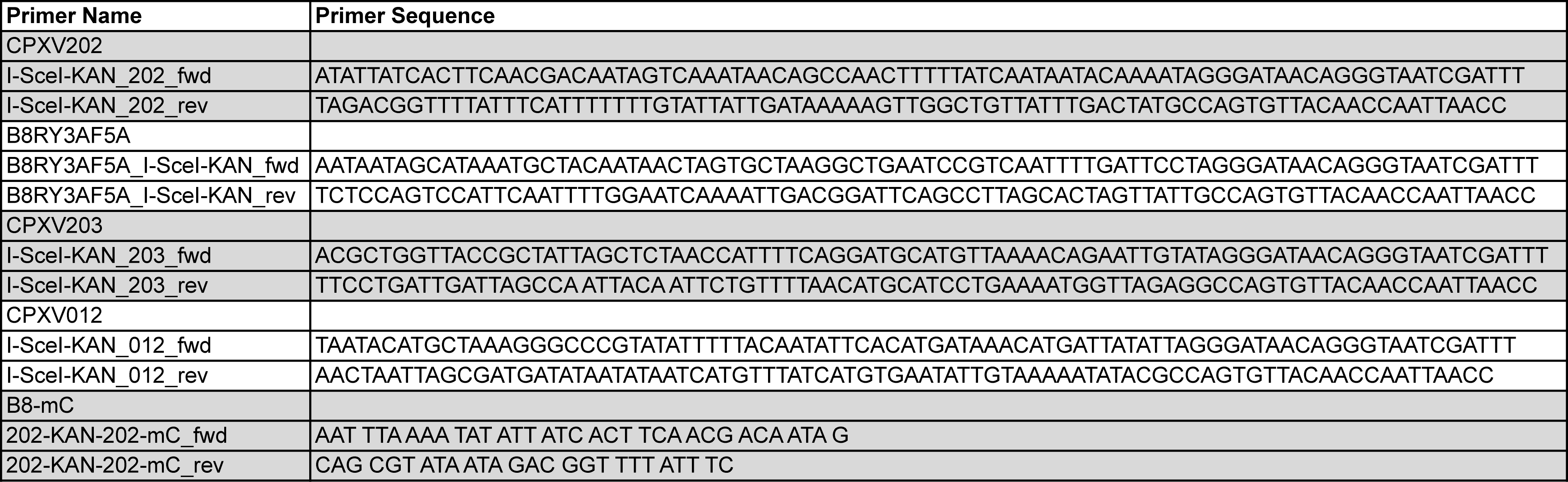

Supplement: S1 Table — (TIF) [file ppat.1006883.s006.tif]

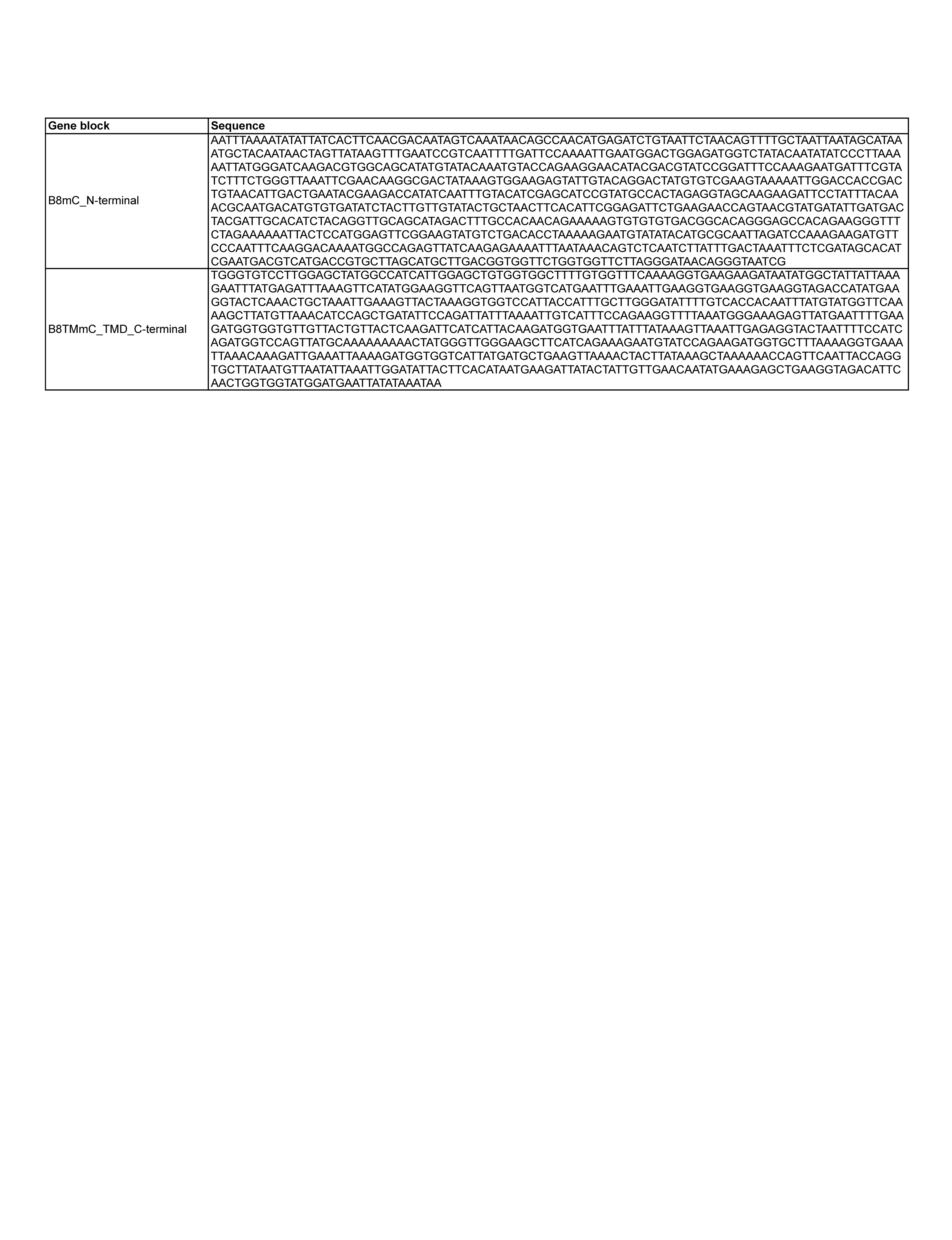

Supplement: S2 Table — (TIF) [file ppat.1006883.s007.tif]

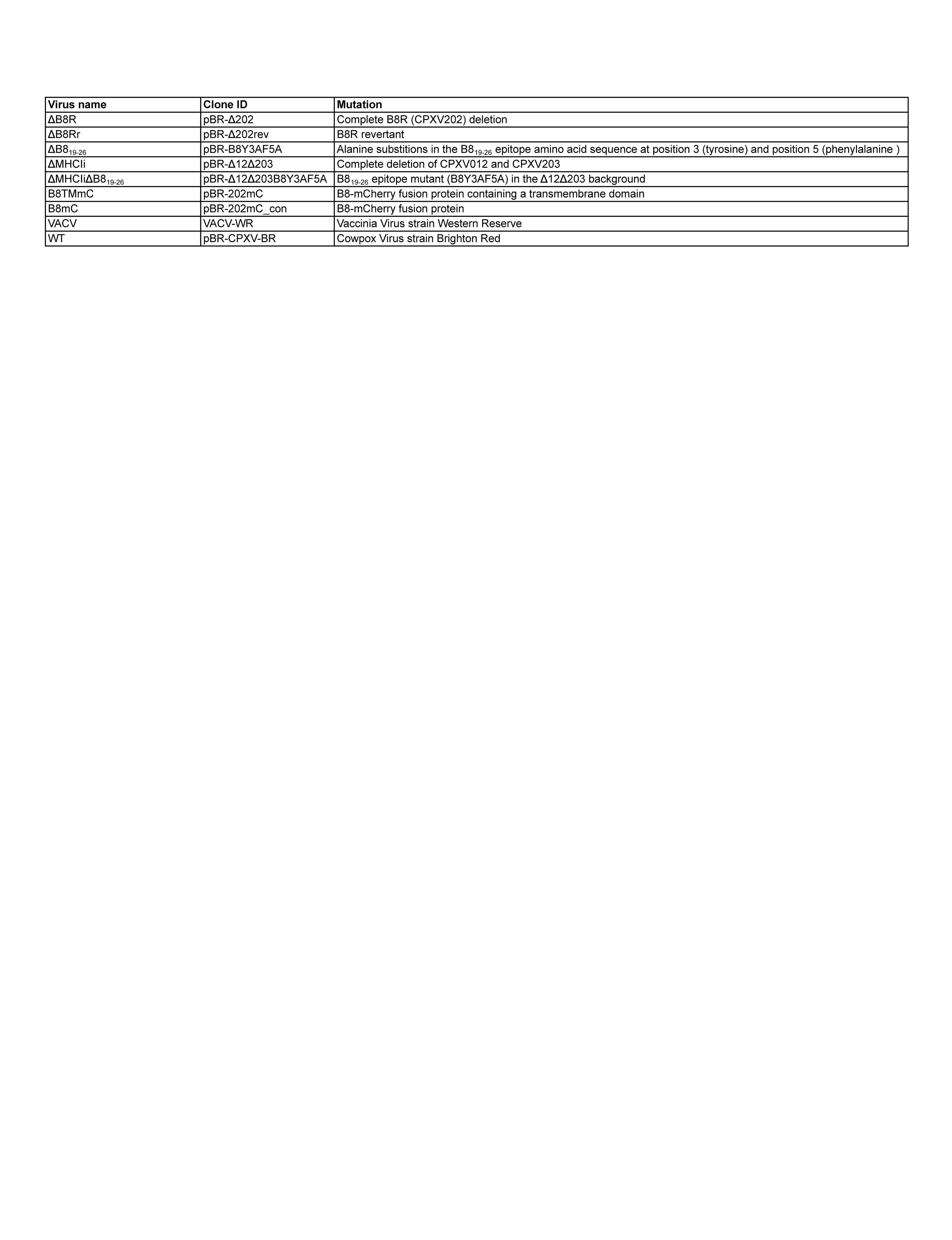

Supplement: S3 Table — (TIF) [file ppat.1006883.s008.tif]
